# Isolation, Characterization and Molecular Identification of Putative Probiotic Bacteria From Red Tilapia Gut

**DOI:** 10.1111/1758-2229.70438

**Published:** 2026-10-21

**Authors:** Remy Ntakirutimana, Mariya Jacob Ashin, Aishath Ali Farhath, Anatole Bigirimana, N. Regina Hershey, Megha Lovejan, Paul Mwangi, M. P. Jijina, Fazeena Fariq, C. Varsha, K. M. Mujeeb Rahiman

**Affiliations:** ^1^ Centre de Recherche en Sciences Naturelles et de l'Environnement (CRSNE) Universite du Burundi Bujumbura Burundi; ^2^ School of Industrial Fisheries Cochin University of Science and Technology Kochi Kerala India; ^3^ Maldives Wetlands Foundation Malé City Maldives; ^4^ Department of Marine Biology, Microbiology and Biochemistry, School of Marine Sciences Cochin University of Science and Technology Cochin Kerala India; ^5^ Department of Biology Univerite du Burundi Bujumbura Burundi; ^6^ Department of Biotechnology Cochin University of Science and Technology Kochi Kerala India

**Keywords:** 16S rRNA gene identification, antimicrobial activity, autochthonous probiotics, gastrointestinal stress tolerance, microbiome, *Oreochromis* sp., safety assessment

## Abstract

The use of probiotics is a promising strategy to enhance fish health and sustainability in aquaculture as alternatives to antibiotics. This study applied a systematic multi‐criteria screening approach to identify autochthonous probiotic candidates from the intestinal microbiota of healthy red tilapia (*Oreochromis* sp.). A total of 26 culturable bacterial isolates were recovered and evaluated for antimicrobial activity against major fish pathogens ( *Aeromonas hydrophila* , *Streptococcus agalactiae* and *Streptococcus iniae* ), safety, tolerance to simulated gastrointestinal conditions, adhesion‐related properties, enzymatic activities and environmental robustness. Only a small subset of isolates exhibited broad‐spectrum antimicrobial activity and satisfied subsequent safety and functional criteria. Based on integrated performance, three isolates were shortlisted for advanced evaluation, of which two were retained for molecular identification. The selected isolates were identified by 16S rRNA gene sequencing as *Pseudomonas aeruginosa* PA4 (PA4) and *Bacillus* sp. BA4P (BA4P). Both strains demonstrated tolerance to gastrointestinal stress, measurable adhesion‐related hydrophobicity, diverse enzymatic activities and stability under varying temperature and salinity conditions. These findings provide a robust framework for screening host‐associated probiotics and support the further evaluation of PA4 and BA4P as potential candidates for sustainable tilapia aquaculture.

## Graphical abstract

A systematic multi‐criteria screening of gut bacteria from healthy red tilapia identified two host‐derived probiotic candidates, *Pseudomonas aeruginosa* PA4 and *Bacillus* sp. BA4P. Both strains exhibited broad‐spectrum antimicrobial activity, safety, gastrointestinal stress tolerance, adhesion‐related properties and functional robustness, supporting their potential for sustainable tilapia aquaculture.

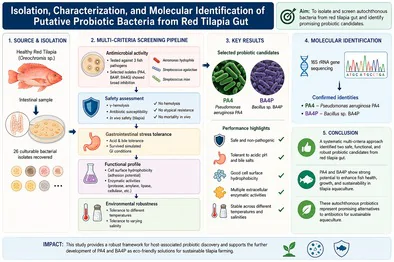

## 1 Introduction

Aquaculture is one of the fastest‐growing food production sectors worldwide, supplying a substantial share of high‐quality animal protein. Tilapia farming plays a central role in meeting this demand, with red tilapia (*Oreochromis* sp.) particularly valued for its rapid growth, environmental adaptability and strong market appeal (FAO 2022; Yuan et al. 2022). However, intensified aquaculture practices often result in disease outbreaks, reduced growth performance and environmental impacts resulting from excessive and inappropriate antibiotic use (Lin et al. 2020; Lorgen‐Ritchie et al. 2023).

Tilapia possess a relatively simple digestive system, which limits their ability to break down complex feed components and often results in suboptimal feed conversion ratios (FCR) (Ng and Koh 2017). Furthermore, intensive farming increases susceptibility to opportunistic pathogens such as *Streptococcus agalactiae* , *Aeromonas* spp. and *Vibrio* spp. (Amal et al. 2018). The conventional use of antibiotics to control infections has contributed to the emergence of antimicrobial resistance (AMR) and ecological disturbances, thereby underscoring the urgent need for sustainable alternatives to antibiotic‐based disease management (Done et al. 2015).

To address these constraints, probiotics have gained attention as a sustainable intervention. FAO/WHO guidelines defined probiotics as live microorganisms that, when administered in adequate amounts, confer health benefits to the host (Morelli and Capurso 2012). Their beneficial effects in aquaculture include competitive exclusion of pathogens, reinforcement of gut barrier integrity, modulation of immune responses and production of digestive enzymes (Ringø 2020; Ringø et al. 2020). Commonly applied probiotics include lactic acid bacteria (*Lactobacillus*, *Enterococcus*), *Bacillus* spp. and yeasts such as *Saccharomyces cerevisiae* , which have been reported to improve feed utilization, disease resistance and stress tolerance in fish (Dawood 2021). For example, dietary *Bacillus subtilis* enhanced nutrient absorption and gut microbiota balance in tilapia, resulting in improved growth (Liu et al. 2021), while *Lactobacillus plantarum* stimulated innate immunity and reduced mortality following *Aeromonas hydrophila* challenge (Gewaily et al. 2021). Similarly, *S. cerevisiae* supplementation has been shown to promote gut health and enhance immune responses in farmed fish (Abdel‐Tawwab et al. 2010).

Despite these promising benefits, probiotic performance is highly dependent on host specificity, as microbial interactions within the gut are influenced by host physiology and environmental conditions (Abdul Manan 2025). Commercially available probiotics are often derived from non‐fish sources, and their efficacy in red tilapia remains insufficiently studied. By contrast, host‐derived probiotics, isolated directly from the gut microbiota of the target species, are naturally adapted to the host's intestinal environment. Such strains are more likely to colonize effectively, compete with pathogens and enhance digestive enzyme activity (Banerjee and Ray 2017; Beck et al. 2015). Indeed, gut‐derived *Bacillus* and *Lactobacillus* isolates have been shown to produce exogenous enzymes such as amylase, protease and lipase, while also exhibiting antimicrobial activity against fish pathogens (Standen et al. 2016).

Recent literature has reinforced the expanding interest in aquaculture probiotics. A 2025 bibliometric review reported a rapid rise in publications focusing on probiotics for growth performance, disease resistance and feed utilization, with Asia leading the research output (Previti et al. 2025). Moreover, studies on *Bacillus* spp. in red tilapia have highlighted improvements in survival, blood profiles and reproductive performance, while novel isolates such as *Staphylococcus edaphicus* have shown potential probiotic properties (Rahayu et al. 2024). Beyond their applied relevance for aquaculture, host‐associated bacteria such as these offer a tractable system for understanding how gut microorganisms acquire and express functional traits, antimicrobial metabolite production, extracellular enzyme secretion and stress tolerance in response to host and environmental selective pressures, thereby contributing to a broader understanding of microbial functional ecology in aquatic systems. These findings emphasize the need for species‐specific probiotic discovery to support sustainable aquaculture.

In this context, the present study was designed with the following objectives:

- To isolate putative probiotic bacteria from the intestinal microbiota of healthy red tilapia.
- To characterize and screen isolates for core probiotic properties.
- To identify selected isolates using molecular techniques, 16S rRNA gene sequencing.

## 2 Materials and Methods

Figure 1 illustrates the systematic, multi‐phase workflow employed for the isolation, screening and characterization of autochthonous probiotics from red tilapia (*Oreochromis* sp.) following Hasan and Banerjee (2020). The pipeline progresses from the initial collection and acclimation of healthy host specimens to the rigorous two‐tier safety assessment and functional profiling of microbial isolates. By integrating in vitro stress tolerance assays with in vivo host tolerability trials and concluding with 16S rRNA molecular identification, this standardized framework ensures the selection of naturally adapted, non‐pathogenic and functionally robust probiotic candidates specifically tailored for aquaculture applications.

**FIGURE 1:**
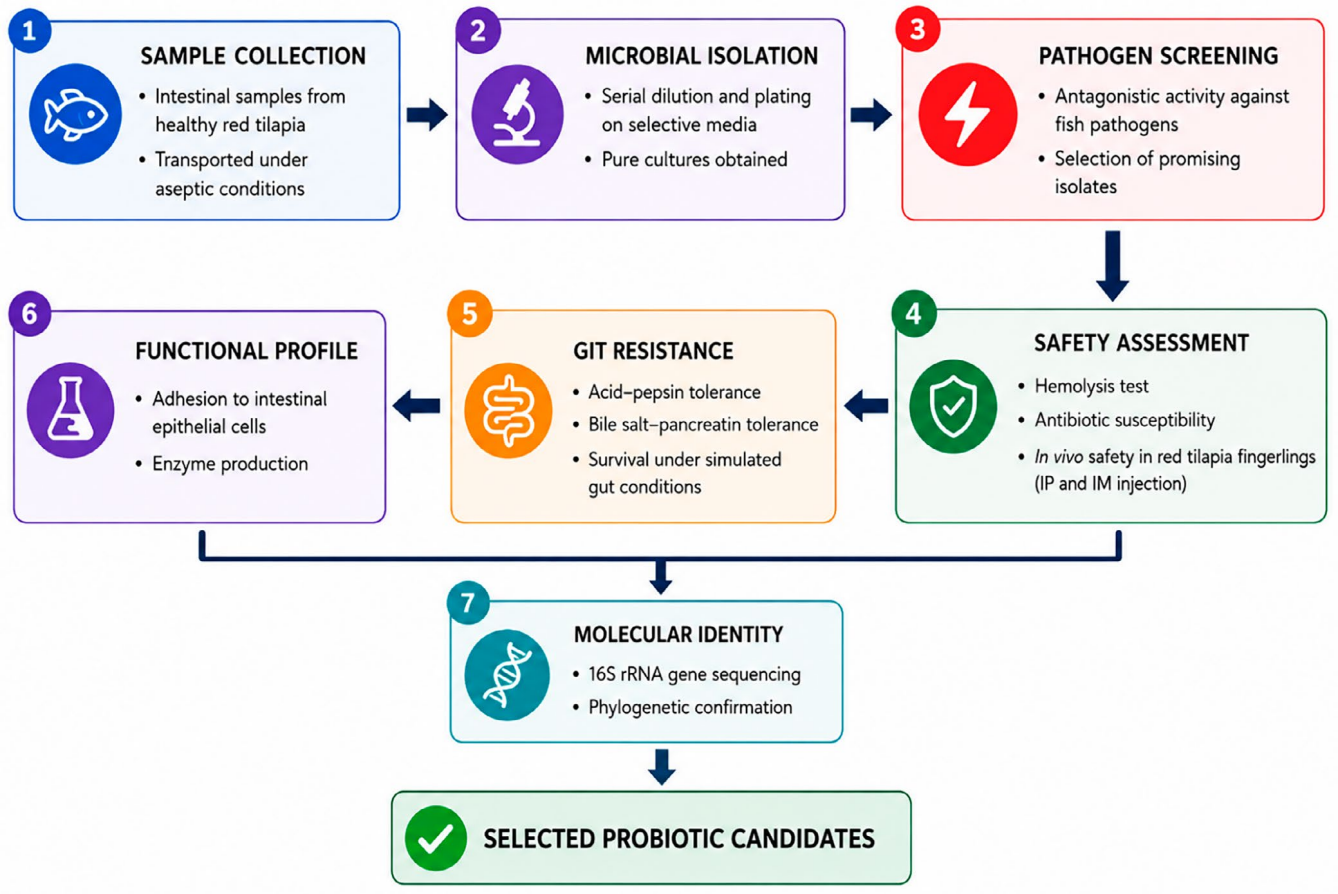
Experimental workflow for the isolation, screening and identification of probiotic candidates from red tilapia gut. GIT, gastrointestinal tract.

### 2.1 Sample Collection

Red tilapia (*Oreochromis* sp.) were collected from an earthen pond at a commercial aquaculture farm in Thrissur, Kerala, India, where probiotics had not previously been used as a feed supplement and transported live to the laboratory of the School of Industrial Fisheries, Cochin University of Science and Technology (CUSAT) in aerated plastic bags containing pond water. Upon arrival, the fish were acclimated for 48 h in 500‐L fibreglass tanks under continuous aeration, with dissolved oxygen maintained at 6–7 mg/L, and fed a standard commercial diet containing 30% crude protein. Following acclimation, six apparently healthy adult red tilapia weighing 150–200 g were randomly selected for intestinal sampling (Ganguly et al. 2019). Euthanasia was performed according to ethical guidelines, using clove essential oil at a concentration of 300 mg/L (Bona et al. 2024; Mitjana et al. 2014) until complete cessation of opercular movement. To ensure aseptic conditions during dissection, each fish was surface sterilized with 70% ethanol and then rinsed thoroughly with sterile phosphate‐buffered saline (PBS, pH 7.3) (Yao et al. 2024). The abdominal cavity was then aseptically opened using sterile surgical instruments, taking particular care to avoid contamination with external epithelium or visceral contents. The entire intestinal tract was carefully excised, beginning with the pyloric ceca and extending approximately 1 cm anterior to the anal orifice. The excised intestines were immediately placed in sterile Petri dishes and washed three times with PBS to remove residual digesta and transient microbial populations (Chauhan and Singh 2019a). After blotting excess moisture with sterile filter paper, the length and wet weight of each intestinal sample were measured to standardize subsequent microbial analyses. The cleaned intestinal tissues were then homogenized in PBS using a pre‐sterilized mortar and then subjected to vigorous vortexing to ensure complete dissociation of microorganisms present in the mucosa. This homogenized intestinal suspension served as the primary inoculum for subsequent microbial isolation procedures.

### 2.2 Gut Microbiota Culture and Colony Selection

After preparation of intestinal homogenates, a 10‐fold serial dilution (10^−1^ to 10^−10^) was performed in sterile 0.9% (w/v) saline to achieve optimal colony separation. From each dilution, 100 μL aliquots were plated in triplicate onto non‐selective and selective media: tryptone soy agar (TSA; HiMedia, India), nutrient agar (NA), Bacillus agar and cetrimide agar, using sterile L‐spreaders. The plates were incubated aerobically at 37°C for 24–48 h (Yao et al. 2024). From plates containing well‐separated colonies, distinct morphotypes were selected based on size, shape, elevation, margins, surface texture, opacity and pigmentation (Rodrigues et al. 2022). Potentially representative colonies of each type were subcultured onto fresh plates of the same medium and incubated under identical conditions until axenic cultures were obtained. Practically, colonies were observed under oblique lighting. Colony diameter was measured using a ruler by measuring the colony diameter in two perpendicular directions and then calculating the average (Su et al. 2012). Each isolate was assigned a unique code linking the medium and the colony serial number on the medium.

### 2.3 Pathogen Inhibition Test

The antimicrobial potential of all isolates was evaluated against three significant aquaculture pathogens: *Streptococcus agalactiae* , *Streptococcus iniae* and *Aeromonas hydrophila* obtained from the Central Marine Fisheries Research Institute (CMFRI), Kochi, India. The assay was conducted using a Mueller‐Hinton agar (MHA, HiMedia, India) well diffusion method to assess the antimicrobial effect (Thi et al. 2023). Overnight cultures of each pathogen were adjusted to 1 × 10^6^ CFU/mL by suspension in sterile saline to ensure consistent inoculum density across tests. MHA plates were inoculated with standardized pathogen lawns ( *S. agalactiae* , *S. iniae* , *A. hydrophila* ; ~10^8^ CFU/mL) using sterile swabs. After 10 min drying, 5 mm wells were aseptically cut and filled with 50 μL of the corresponding isolate suspension (Mujeeb Rahiman et al. 2010). Plates were incubated at 37°C for 24 h, and zones of inhibition (mm) were measured. Sterile saline in wells served as a negative control; all tests were performed in triplicate. Isolates demonstrating consistent inhibition zones were considered positive for antimicrobial activity (Govindaraj et al. 2021). Particular attention was given to isolates showing broad‐spectrum inhibition (effective against ≥ 3 pathogens) as these were prioritized for subsequent probiotic characterization.

### 2.4 Safety Assessment

A two‐tier assessment was performed, in vitro assays (haemolytic activity and antibiotic susceptibility) and an in vivo safety evaluation in red tilapia with post‐administration monitoring. These assays were used to screen intrinsic pathogenicity and host tolerability; detailed methods follow.

#### 2.4.1 Haemolytic Activity

The haemolytic potential of the probiotic candidates was assessed as a primary safety screen to exclude isolates with pathogenic traits. Following Jang et al. (2023), overnight cultures were spot‐inoculated on Blood agar (HiMedia, India) and incubated aerobically at 37°C for 48 h. Following incubation, plates were examined for zones of haemolysis around the colonies, categorized as complete (β‐haemolysis: clear, translucent zone), partial (α‐haemolysis: greenish discoloration) or absent (γ‐haemolysis: no change).

#### 2.4.2 Antibiotic Susceptibility

Phenotypic susceptibility was determined by the Kirby–Bauer disc diffusion method on Mueller Hinton agar (MHA) (HiMedia, India) following (Muñoz‐Atienza et al. 2013). Lawn cultures were prepared, and discs from clinically relevant classes, namely Vancomycin, Streptomycin, Gentamicin, Tetracycline, Erythromycin, Ceftazidime, Chloramphenicol, Carbenicillin, Polymyxin‐B, Cefepime and Ciprofloxacin, were applied. Plates were incubated at 37°C for 24 h. Zone diameters (mm) were measured and reported.

#### 2.4.3 In Vivo Safety in Red Tilapia

Cell suspensions were prepared from overnight cultures grown in tryptic soy broth (TSB), washed twice with sterile 0.85% saline and adjusted to a final concentration of approximately 1 × 10^8^ cells mL^−1^. Ten red tilapia (*Oreochromis* sp.) fingerlings (13 ± 1.25 g) per tank were administered 0.1 mL fish^−1^ of the respective bacterial suspension via intraperitoneal (i.p.) and intramuscular (i.m.) injection, while control fish received sterile saline only (Lahtinen et al. 2009; Lin et al. 2025). Each treatment was conducted in triplicate, resulting in a total of 30 fingerlings per treatment. Fish were maintained in aerated, dechlorinated freshwater at 28°C ± 1°C, fed a commercial diet (GROWFIN, Growel, India) and subjected to approximately 30% daily water exchange. Animals were monitored for 14 days for mortality and clinical abnormalities, including behavioural changes, feed intake, external lesions and injection‐site reactions (Guimarães et al. 2022). The absence of adverse effects compared with the control group was considered indicative of in vivo safety. All experimental procedures were performed in accordance with institutional animal ethics and care guidelines.

To further substantiate the in vivo safety of the tested isolates and to detect any sub‐clinical physiological disturbances, plasma biochemical indices associated with hepatic and renal function were evaluated (Ntakirutimana et al. 2025). Blood samples were collected from the caudal vein of six fish per treatment at the end of the experimental period using sterile syringes without anticoagulant. Samples were allowed to clot at room temperature for 30 min and subsequently centrifuged at 3000 *g* for 10 min at 4°C to obtain plasma. Plasma levels of glutamate oxaloacetate transaminase (GOT), glutamate pyruvate transaminase (GPT), alkaline phosphatase (ALP), creatinine (CRE) and blood urea nitrogen (BUN) were quantified using a DRI‐CHEM NX500 dry‐chemistry analyzer (Fujifilm, Germany) in accordance with the manufacturer's instructions.

### 2.5 Resistance to Gastrointestinal Simulated Conditions

#### 2.5.1 Bile Salt and Pancreatin Tolerance

Tolerance to combined bile salts and pancreatin was evaluated using a modified simulated intestinal fluid (SIF) assay designed to mimic intestinal conditions (Treven et al. 2024). Overnight bacterial cultures were harvested by centrifugation, washed twice with sterile 0.85% (w/v) phosphate‐buffered saline and resuspended in sterile Nutrient Broth. The cell density of each suspension was adjusted to an initial optical density (OD_600_) of approximately 0.5, corresponding to about 10^6^–10^7^ CFU mL^−1^ in the logarithmic growth phase. Simulated intestinal fluid was prepared by supplementing sterile Nutrient Broth with 0.3% (w/v) bile salts and 1 mg mL^−1^ pancreatin (HiMedia, India), and the pH was adjusted to 8.0 using sterile 1 N NaOH to approximate intestinal conditions. For each isolate, 10 mL aliquots of the prepared SIF and unsupplemented Nutrient Broth (control) were inoculated at 1% (v/v), gently mixed and incubated at 37°C under static conditions. Bacterial tolerance was monitored by measuring optical density at 600 nm at 0, 1, 4 and 6 h using a spectrophotometer, with uninoculated broth serving as the blank. All assays were conducted using three independent biological replicates, each with two technical replicates. Negative control tubes were maintained throughout the experiment to confirm sterility of media and reagents (Siangpro et al. 2023).

Survival was quantified as percentage OD_600_ retention, calculated as:

$$\text{Survival}\;\left(\%\right)=\left(\frac{{\mathrm{OD}}_{600,t}}{{\mathrm{OD}}_{600,0}}\right)\times 100.$$

#### 2.5.2 Acid and Pepsin Tolerance

Acid–pepsin tolerance was evaluated following the general framework of Coulibaly et al. (2023), with modifications to align with the conditions used for the bile–pancreatin assay. Logarithmic‐phase cultures were harvested when they reached OD_600_ ≈ 0.5 (corresponding to approximately 10^6^–10^7^ CFU mL^−1^). Cells were collected by centrifugation (15 min, 4°C), washed twice in sterile 0.85% (w/v) PBS and resuspended in sterile Nutrient Broth (NB). Simulated gastric conditions were prepared by adjusting NB to pH 2.0 and supplementing with 3 mg mL^−1^ pepsin. For each isolate, 10 mL aliquots of acidified NB with acid‐pepsin were dispensed into sterile 50 mL tubes and inoculated at 1% (v/v) with the standardized OD_600_ ≈ 0.5 cell suspension, mixed gently and incubated at 37°C. The tolerance was monitored by measuring OD_600_ at 0, 1, 4 and 6 h, using uninoculated medium as blank. The OD_600_ at 0 h was used as the baseline relative to which survival was expressed as:

$$\text{Survival}\;\left(\%\right)=\left(\frac{{\mathrm{OD}}_{600,t}}{{\mathrm{OD}}_{600,0}}\right)\times 100.$$

### 2.6 Growth Conditions of Selected Isolates

Environmental tolerance of selected isolates was evaluated across temperature and salinity to mirror different tilapia production conditions following (Nayak and Mukherjee 2011). Temperature optimization study was conducted across a gradient from 10°C, 20°C, 35°C and 40°C, encompassing both ambient aquaculture conditions and potential fermentation temperatures. Isolates were cultured in triplicate in nutrient broth, with growth kinetics monitored spectrophotometrically (OD_600_) after 24 h. The temperature yielding the highest specific growth rate (*μ*_max_) during the logarithmic phase was designated as optimal, while the range between temperatures maintaining ≥ 80% of maximal growth was recorded as the functional tolerance window. Salinity tolerance was tested in media containing 0%, 2% and 3% NaCl, incubated at the isolate's optimum temperature. Growth, OD_600_, after 24 and 48 h was recorded; isolates maintaining ≥ 70% growth at 3% NaCl were deemed osmotolerant, relevant for brackish applications. All assays used triplicate biological replicates with appropriate media controls.

### 2.7 Motility Test

Motility test was done by both methods hanging‐drop wet mount (Jain et al. 2020) and semi‐solid agar stab (Tittsler and Sandholzer 1936) in a single workflow. Fresh TSA colonies were suspended in sterile 0.85% NaCl to a light turbidity; a hanging drop was prepared on a cavity slide and examined immediately at 400–1000× to detect directional displacement (true motility) versus Brownian motion (non‐motile), scoring at least three fields across three preparations. In parallel, semi‐solid motility medium was sterilized and inoculated from a single colony by one central vertical stab to ~2/3 depth. Tubes were incubated at 37°C for 24–48 h (up to 48 h for slow growers) and interpreted as motile when diffuse/umbrella‐like growth (±red TTC diffusion) extended away from the stab line, or non‐motile when growth remained confined to the stab. Results were accepted when both methods agreed; discrepant outcomes were retested.

### 2.8 Gram's Staining of the Isolates

Gram staining was performed using the standard four‐reagent method (Smith and Hussey 2019). Briefly, thin smears from fresh TSA colonies were prepared on grease‐free slides, air‐dried and heat‐fixed. Smears were flooded with 1% crystal violet for 60 s, rinsed with distilled water, covered with Gram's iodine for 60 s, rinsed and decolorized with 95% ethanol for 10–15 s until runoff was pale. Slides were immediately rinsed and counterstained with 0.5% safranin for 30 s, rinsed, air‐dried and examined under oil immersion (1000×). Purple cells were recorded as Gram‐positive and pink cells as Gram‐negative; cell morphology (rod/coccus), arrangement and any pleomorphism were noted.

### 2.9 Endospore Staining

Endospore formation was assessed for the candidate using the Schaeffer–Fulton method as described by the American Society of Microbiology (Hussey and Zayaitz 2007). Thin smears from fresh Nutrient Agar colonies were prepared on grease‐free slides, air‐dried and heat‐fixed. Smears were placed over gentle steam and flooded with 0.5% (w/v) malachite green (aqueous, HiMedia, India) for 5 min, maintaining a wet surface throughout. Slides were removed, cooled briefly and rinsed thoroughly with distilled water for 30–60 s to decolorize vegetative cells. Preparations were counterstained with 0.5% (w/v) safranin for 30–60 s, rinsed, air‐dried and examined under oil immersion (1000×). Endospores appeared green (intracellular or free), while vegetative cells appeared red/pink. The *Pseudomonas*‐like candidate was not expected to form endospores and served as a negative biological reference. All assays were performed in triplicate.

### 2.10 Production of Siderophores

Siderophore secretion was assessed on Chrome Azurol S (CAS) agar (HiMedia, India) prepared according to the manufacturer's instructions and poured (~20 mL/plate) under aseptic conditions. Overnight TSB cultures of the probiotic candidates were spot‐inoculated (5–10 μL) or lightly streaked onto CAS plates and incubated at 37°C for 24–48 h. Development of orange/yellow halos against the blue background was recorded as siderophore‐positive; halo diameters were measured with digital callipers and reported as mean ± SD from 3 biological replicates. Each batch included an uninoculated plate (background control) and a known siderophore producer (positive control). Where strong colony pigmentation interfered with interpretation, readings were repeated on freshly poured plates with thinner inocula.

### 2.11 Cell Surface Hydrophobicity Test

Cell surface hydrophobicity of the probiotic candidates was evaluated as an indicator of their potential adhesive capacity to host epithelial surfaces, following (Mirzabekyan et al. 2023) using xylene as the hydrophobic solvent.

The isolates were grown in nutrient broth at 37°C for 24 h. Logarithmic‐phase cultures were harvested by centrifugation at 4000 *g* for 10 min at 4°C, and the pellets were washed twice with sterile phosphate‐buffered saline (PBS, pH 7.2). The cells were then resuspended in PBS and the suspension adjusted to an optical density of 0.5 at 600 nm (*A*_*o*_).

An aliquot of 10 mL of the standardized cell suspension was transferred into sterile glass tubes, and 2 mL of xylene was gently added. The mixture was vortexed vigorously for 1 min to facilitate interaction between bacterial cells and the solvent, and then incubated at 37°C for approximately 2 h until two distinct phases were formed. After phase separation, the aqueous (lower) phase was carefully collected without disturbing the xylene phase, and its absorbance was measured again at 600 nm (*A*_*f*_).

Cell surface hydrophobicity was expressed as the percentage decrease in absorbance of the aqueous phase by the following equation:

$$\text{Hydrophobicity}\;\left(\%\right)=\frac{\left(A_{0}-A_{f}\right)}{A_{0}}\times 100,$$

where *A*_*o*_ is the initial absorbance of the bacterial suspension in PBS before mixing with xylene and *A*_*f*_ is the absorbance of the aqueous phase after incubation with xylene.

### 2.12 Biochemical and Enzymatic Profiling of the Isolates

#### 2.12.1 Oxidase and Catalase Production

A new culture was prepared for the isolates by inoculating each of them on an appropriate growth medium (TSA) and allowing it to grow for 24 h at the above recommended temperature. The oxidase test was done using the oxidase test discs (HiMedia) according to the manufacturer. A sterilized inoculating loop was brought into contact with a single colony and the loop containing the bacterial colony was gently touched to the disc. The presence of the oxidase enzyme in the isolates caused a colour change on the filter paper. A positive result was indicated by the appearance of a dark blue or purple colour within 10–30 s. The presence of catalase was demonstrated by the quick formation of bubbles resulting from the liberation of molecular oxygen after introducing a 5% H_2_O_2_ solution onto a bacterial colony situated on a glass slide (Mohamad et al. 2020).

#### 2.12.2 Protease Test

Using a sterile inoculation loop, bacterial isolates were streaked on Casein hydrolysate agar (g/l: enzymatic casein hydrolysate 5, beef infusion 150, peptic digestion of animal tissue 5, yeast autolysate 1.5, sodium phosphate 2.5, sodium chloride 5, agar 15, pH 7.8 ± 0.2; Hi‐media, India) supplemented with 10% (v/v) UHT sterile skim milk (Nakharuthai et al. 2023). After incubation at 37°C of the plates for 24 h, the activity of the protease was evidenced by the appearance of clear zones called ‘clearance halos’ around or under the bacterial colonies, indicating the degradation of proteins in the environment. These areas indicate the presence of protease enzymes.

#### 2.12.3 Cellulase Production

The cellulase test was carried out according to the method described by (Kasana et al. 2008). Culture of bacterial strains was performed on carboxymethylcellulose (CMC) agar plates. After 24 h of incubation at 37°C, the plates were flooded with Congo red solution for 5 min. The appearance of a clear and distinct zone around the colony indicates cellulase‐producing microbial colonies.

#### 2.12.4 Phosphate Solubility Test

Phosphate solubilization was evaluated on Pikovskaya's (PVK) agar (HiMedia, India) prepared per manufacturer's instructions (dehydrated PVK reconstituted and sterilized; ~20 mL/plate) with the following composition (g L^−1^): yeast extract 0.5, dextrose 10, tricalcium phosphate (TCP) 5, (NH_4_)_2_SO_4_ 0.5, KCl 0.2, MgSO_4_·7H_2_O 0.1, MnSO_4_·H_2_O 0.0001, FeSO_4_·7H_2_O 0.0001, agar 15; pH 7.0 ± 0.2. Overnight cultures were streaked onto PVK and incubated at 37°C for 48–72 h (extended to 5 days if halos were faint). Clear halos around colonies were recorded as inorganic phosphate solubilization. Assays were performed in triplicate.

#### 2.12.5 Indole Production

Bacteria with the ability to produce the enzyme tryptophanase are said to be indole positive. Indeed, tryptophanase hydrolyses the amino acid tryptophan into indole, pyruvic acid and ammonia (Melander et al. 2014). This test was performed following MacWilliams (2016) protocol. The tryptone broth tube was inoculated with a tiny amount of the isolate and incubated at 37°C for 24–48 h. Following incubation, five drops of the Kovács reagent were immediately added to the tube. In the reagent layer above the middle, a few seconds after the reagent is introduced, a pink‐to‐red colour known as a ‘cherry red ring’ appears for a positive indole test. The reagent layer will remain yellow or barely hazy if a culture is indole negative.

#### 2.12.6 Lipase Activity Assay

Lipase activity of the bacterial isolates was assessed following Ertuǧrul et al. (2007), using tributyrin agar prepared and autoclaved at 121°C for 15 min at 15 psi. The medium was allowed to cool to approximately 50°C before pouring into sterile Petri dishes under aseptic conditions. Bacterial isolates were cultured overnight in nutrient broth at 37°C with continuous agitation at 150 rpm. A standardized inoculum of each isolate (adjusted to an optical density of 0.5 McFarland standard) was spot‐inoculated onto the surface of the solidified tributyrin agar plates. Plates were incubated aerobically at 37°C for 48 h. Following incubation, the plates were examined for the presence of clear zones around the bacterial growth. The appearance of a transparent halo surrounding the colonies indicated lipase activity due to the hydrolysis of tributyrin. The diameter of the clear zone was measured in millimetres using a digital calliper to quantify the lipase activity. All assays were performed in triplicate to ensure reproducibility and reliability of the results.

#### 2.12.7 Amylase Activity Assay

Amylase activity (Starch hydrolysis) of the bacterial isolates was assessed using starch agar (HiMedia, India) following the manufacturer's instructions. Bacterial isolates were cultured overnight in nutrient broth at 37°C with continuous agitation at 150 rpm. A standardized inoculum of each isolate (adjusted to an optical density of 0.5 McFarland standard) was spot‐inoculated onto the surface of the solidified starch agar plates. Plates were incubated aerobically at 37°C for 48 h. After incubation, the plates were flooded with Gram's iodine solution (1% iodine and 2% potassium iodide) and allowed to stand for 5 min. The presence of a clear zone around the bacterial colonies indicated amylase activity due to the hydrolysis of starch. The diameter of the clear zone was measured in millimetres using a digital calliper to quantify the amylase activity. All assays were performed in triplicate to ensure reproducibility and reliability of the results.

#### 2.12.8 Urea Hydrolysis

Urease activity of probiotic candidates was evaluated on Christensen's urea agar slants (HiMedia, India) following the manufacturer's instructions. The basal composition per litre was: peptone 1.0 g, glucose 1.0 g, NaCl 5.0 g, KH_2_PO_4_ 2.0 g, phenol red 0.012 g and agar 15 g (pH 6.8 ± 0.2). The basal medium was autoclaved (121°C, 15 min), cooled to 50°C–55°C, then aseptically supplemented with filter‐sterilized urea to 2.0% (w/v). While still warm, ~5–7 mL portions were dispensed into sterile tubes and set at an angle to obtain slants. Fresh colonies from TSA were used to lightly streak the surface of each slant (single zig‐zag; no heavy inoculum). Slants were incubated at 37°C and examined at 4, 24 and 48 h. Urease activity was interpreted from the phenol‐red shift (yellow/orange → pink/magenta) and recorded as rapid/strong positive (distinct pink ≤ 4 h), positive (pink by 24 h), weak/delayed (faint pink only by 48 h) or negative (no change).

### 2.13 Molecular Identification of the Isolates

The bacterial isolates were identified by amplification of the 16S rRNA gene using the universal primers described by Weisburg et al. (1991). The primer pair used was 27F (5′‐AGAGTTTGATCMTGGCTCAG‐3′) and 1492R (5′‐TACGGYTACCTTGTTACGACTT‐3′), which amplify an approximately 1500 bp fragment of the bacterial 16S rRNA gene. PCR amplification was carried out in a final reaction volume of 25 μL containing 12.5 μL of 2× PCR Master Mix (Takara Bio Inc., Japan), 1 μL each of forward and reverse primers (10 μM), 1 μL of template DNA (approximately 25 ng) and 9.5 μL of nuclease‐free water. The 2× PCR Master Mix contained Taq DNA polymerase, reaction buffer, MgCl_2_ and dNTPs according to the manufacturer's specifications. No additional PCR additives were used. Amplification was performed in a thermal cycler using an initial denaturation at 95°C for 5 min, followed by 30 cycles of denaturation at 95°C for 1 min, annealing at 59°C for 1 min and extension at 72°C for 2 min, with a final extension at 72°C for 10 min.

The amplified PCR products were resolved by electrophoresis on a 1.5% agarose gel prepared in 1× TAE buffer at 90 V for 40 min, stained with ethidium bromide (EtBr) and visualized under a UV transilluminator. The expected amplicon size was approximately 1500 bp. Successful PCR products were purified using ExoSAP‐IT PCR Product Cleanup Reagent (Applied Biosystems, USA) and subjected to bidirectional Sanger sequencing using the BigDye Terminator v3.1 Cycle Sequencing Kit on an ABI 3500 Genetic Analyzer (Applied Biosystems, USA). Forward and reverse sequences were quality‐checked, trimmed and assembled into consensus sequences. Consensus sequences were compared against the NCBI GenBank database using the BLASTn algorithm for preliminary taxonomic identification. Multiple sequence alignment was performed using MUSCLE, and phylogenetic analysis was conducted using the Maximum Likelihood method implemented in MEGA version 12. The obtained 16S rRNA gene sequences were deposited in the GenBank database under their respective accession numbers.

### 2.14 Data Analysis

Quantitative data were expressed as mean ± standard deviation (SD) of three replicates. Statistical analyses were performed using R version 4.5.2. Differences among groups were evaluated by one‐way analysis of variance (ANOVA), followed by Tukey's HSD post hoc test where appropriate. Statistical significance was considered at *p* < 0.05.

## 3 Results

### 3.1 Isolation and Phenotypic Diversity of Intestinal Bacteria

A total of 26 culturable bacterial isolates were recovered from the intestinal tract of healthy red tilapia following serial dilution and plating on selective and non‐selective media (nutrient agar, tryptone soy agar, Bacillus agar and cetrimide agar). The isolates exhibited marked phenotypic diversity with respect to colony morphology, including differences in size, pigmentation, elevation, surface texture and margin characteristics. Microscopic examination revealed that all isolates were rod‐shaped, with several isolates showing endospore formation (Table S1).

### 3.2 Antimicrobial Activity Against Fish Pathogens

The antimicrobial activity of the isolates was screened against three major fish pathogens ( *A. hydrophila* , *S. agalactiae* and *S. iniae* ). Most isolates (17/26) did not exhibit detectable antagonistic activity, while a limited number inhibited one (5 isolates), two (1 isolate) or all three pathogens (3 isolates) (Table S2, Figure 2A). Based on this preliminary screening, three isolates (PA4, BA4P and BA4G) showing inhibition against three pathogens were selected for further analysis. Quantitative evaluation of these shortlisted isolates revealed significant isolate‐ and pathogen‐specific differences in antagonistic activity (*p* < 0.01). Against *A. hydrophila* and *S. iniae* , PA4 produced the largest inhibition zones, followed by BA4P, whereas BA4G showed significantly lower activity. In contrast, BA4P exhibited the strongest inhibition against *S. agalactiae* , significantly exceeding PA4, while BA4G again showed minimal activity (Figure 2B). The photographs of the well‐diffusion assay on Mueller–Hinton agar (MHA) are presented in Figure 2C. These results confirm distinct antagonistic profiles among the selected isolates, with PA4 and BA4P demonstrating superior and complementary antimicrobial potential.

**FIGURE 2:**
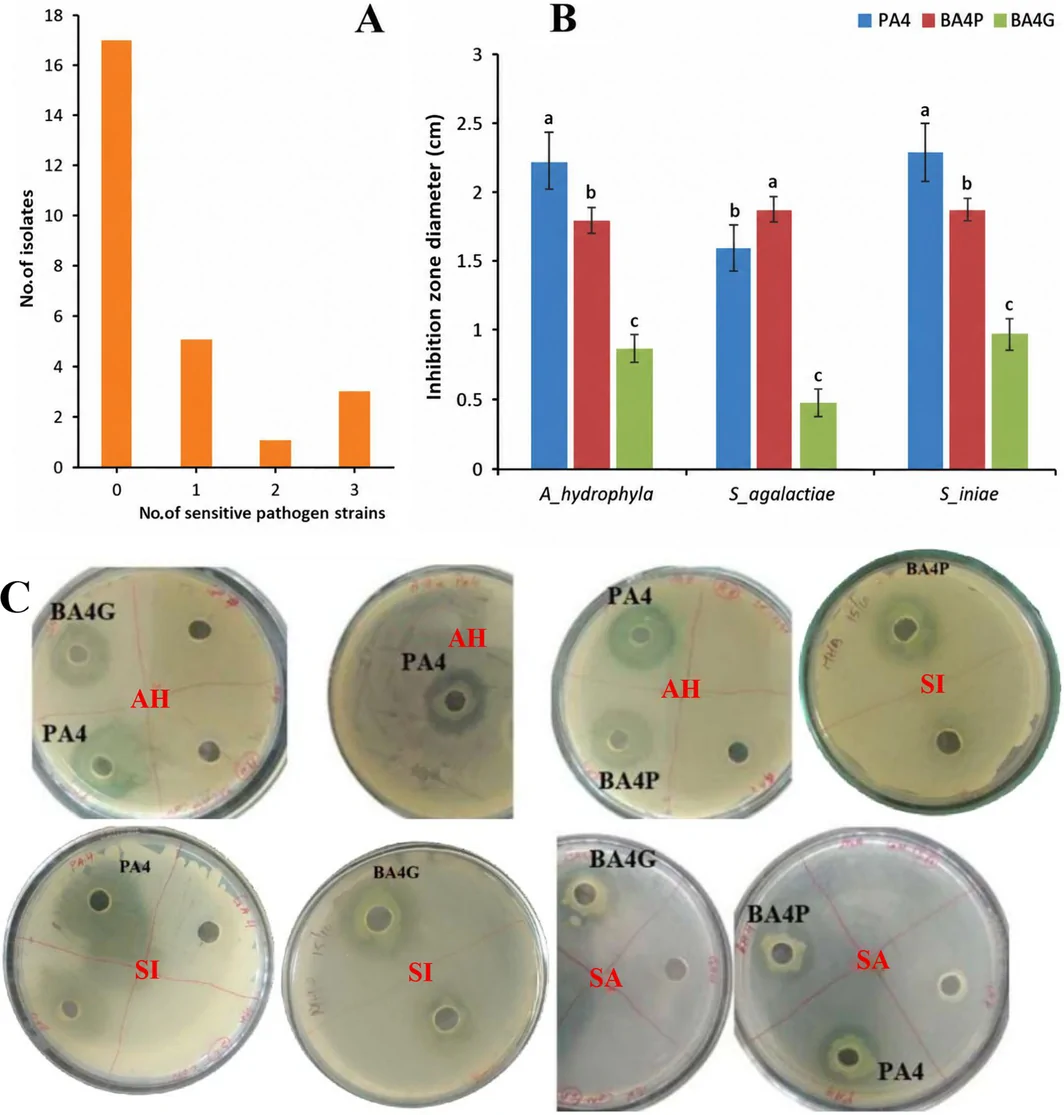
Antimicrobial activity of intestinal bacterial isolates against major fish pathogens. (A) Number of isolates inhibiting 0, 1, 2 or 3 pathogens. (B) Inhibition‐zone diameters of PA4, BA4P and BA4G against *A. hydrophila* , *S. agalactiae* and *S. iniae* . Values are mean ± SD (*n* = 3); error bars represent SD among replicates. (C) Representative photographs of the well‐diffusion assay on Mueller–Hinton agar (MHA); multiple isolates were randomly positioned on individual plates, and only isolates showing inhibition are represented. AH, *A. hydrophila* ; SA, *S. agalactiae* ; SI, *S. iniae* .

### 3.3 Safety Profile of the Candidates

All shortlisted candidates (PA4, BA4P and BA4G) exhibited γ‐haemolysis, indicating the absence of haemolytic activity (Table 1). Survival progression over the experimental period showed stable trends in all treated groups, comparable to the control (Figure 3A). In vivo safety evaluation revealed no treatment‐related mortality following intraperitoneal administration of the isolates. After 14 days, mean survival was 93.33% ± 5.77% for BA4G, 100.00% ± 0.00% for BA4P, 86.67% ± 11.55% for CON and 100.00% ± 0.00% for PA4. Survival rates did not differ significantly among treatments and the control (*p* = 0.33) (Figure 3B). Antibiotic susceptibility profiling showed that all three isolates were broadly susceptible to the tested antibiotics, with comparable inhibition responses across vancomycin, streptomycin, gentamicin, tetracycline, erythromycin, cephalosporins, chloramphenicol, carbenicillin, polymyxin‐B and ciprofloxacin, and no isolate displayed an atypical resistance pattern (Table 1, Figure 3C). Plasma biochemical analysis further supported the safety of the candidate isolates, as hepatic and renal function markers remained within normal physiological ranges (Table 2). Activities of GOT and GPT in treated fish were comparable to the control, while ALP, creatinine and blood urea nitrogen levels remained below detectable or threshold limits across all groups. Collectively, these findings indicate that PA4, BA4P and BA4G are non‐haemolytic, antibiotic‐susceptible and physiologically safe for red tilapia under the tested conditions.

**TABLE 1:** Haemolytic activity and antibiotic sensitivity of selected isolates (mean ± SD in cm).

Antibiotics	BA4G	BA4P	PA4
Haemolytic activity	γ	γ	γ
Vancomycin	2.83 ± 0.35	2.73 ± 0.49	2.80 ± 0.17
Streptomycin	2.77 ± 0.55	2.73 ± 0.40	2.83 ± 0.50
Gentamicin	2.47 ± 0.35	2.70 ± 0.20	2.63 ± 0.50
Tetracycline	2.60 ± 0.35	2.97 ± 0.32	2.63 ± 0.47
Erythromycin	2.77 ± 0.40	2.97 ± 0.47	2.90 ± 0.26
Ceftazidime	2.80 ± 0.36	2.77 ± 0.38	2.63 ± 0.23
Chloramphenicol	2.83 ± 0.55	2.33 ± 0.32	2.47 ± 0.25
Carbenicillin	2.67 ± 0.15	2.63 ± 0.15	2.53 ± 0.47
Polymyxin‐b	3.10 ± 0.26	2.83 ± 0.46	2.73 ± 0.23
Cefepime	2.70 ± 0.44	2.57 ± 0.50	2.80 ± 0.61
Ciprofloxacin	2.63 ± 0.50	2.77 ± 0.21	3.00 ± 0.10

**FIGURE 3:**
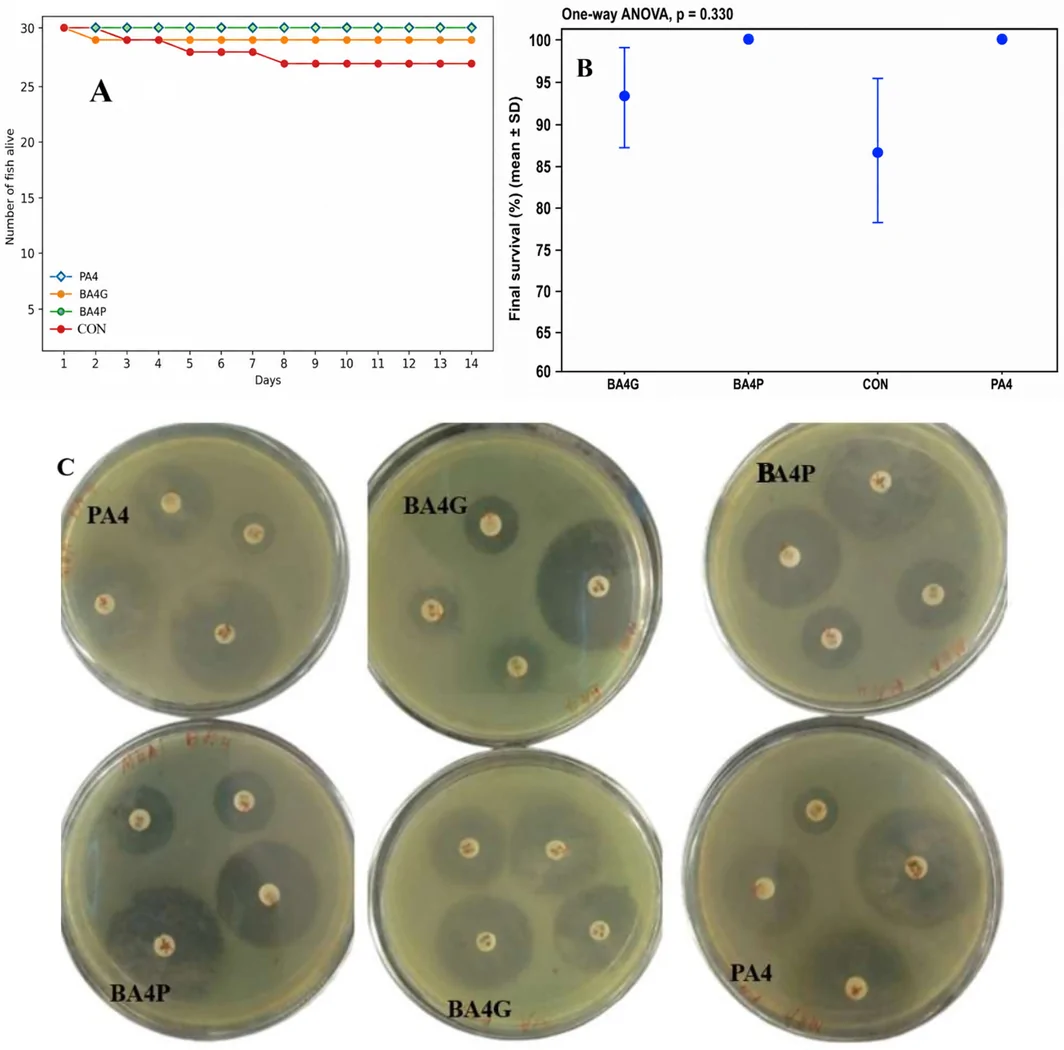
In vivo and in vitro safety assessment of probiotic candidates PA4, BA4P and BA4G in red tilapia fingerlings following intraperitoneal and intramuscular administration. (A) Survival dynamics over the 14‐day observation period for the control (CON) and treated groups. (B) Final survival rate (%) at Day 14 expressed as mean ± SD. (C) Some illustrative MHA plates of the susceptibility of three selected probiotic candidates from red tilapia gut on antibiotics. Data are based on three replicates (*n* = 3).

**TABLE 2:** Blood plasma biochemical parameters of red tilapia IP & IM injected with isolates.

Parameter	Control	BA4G	BA4P	PA4
GOT (U/L)	31.12 ± 2.18	32.31 ± 3.13	31.14 ± 2.22	31.11 ± 1.10
GPT (U/L)	22.12 ± 2.21	20.13 ± 1.18	20.51 ± 1.32	19.22 ± 1.22
ALP (U/L)	< 50.00	< 50.00	< 50.00	< 50.00
CRE (mg/dL)	< 0.20	< 0.20	< 0.20	< 0.20
BUN (mg/dL)	< 5.00	< 5.00	< 5.00	< 5.00

Abbreviations: ALP, alkaline phosphatase; BUN, blood urea nitrogen; CRE, creatinine; GOT, glutamate oxaloacetate transaminase; GPT, glutamate pyruvate transaminase.

### 3.4 Cell Surface Hydrophobicity

Cell surface hydrophobicity of PA4, BA4P and BA4G was assessed as an indicator of adhesive potential. All three isolates exhibited measurable hydrophobicity, with isolate‐specific differences in percentage OD_600_ reduction (Figure 4A). The comparative hydrophobicity profiles are presented in Figure 4B.

**FIGURE 4:**
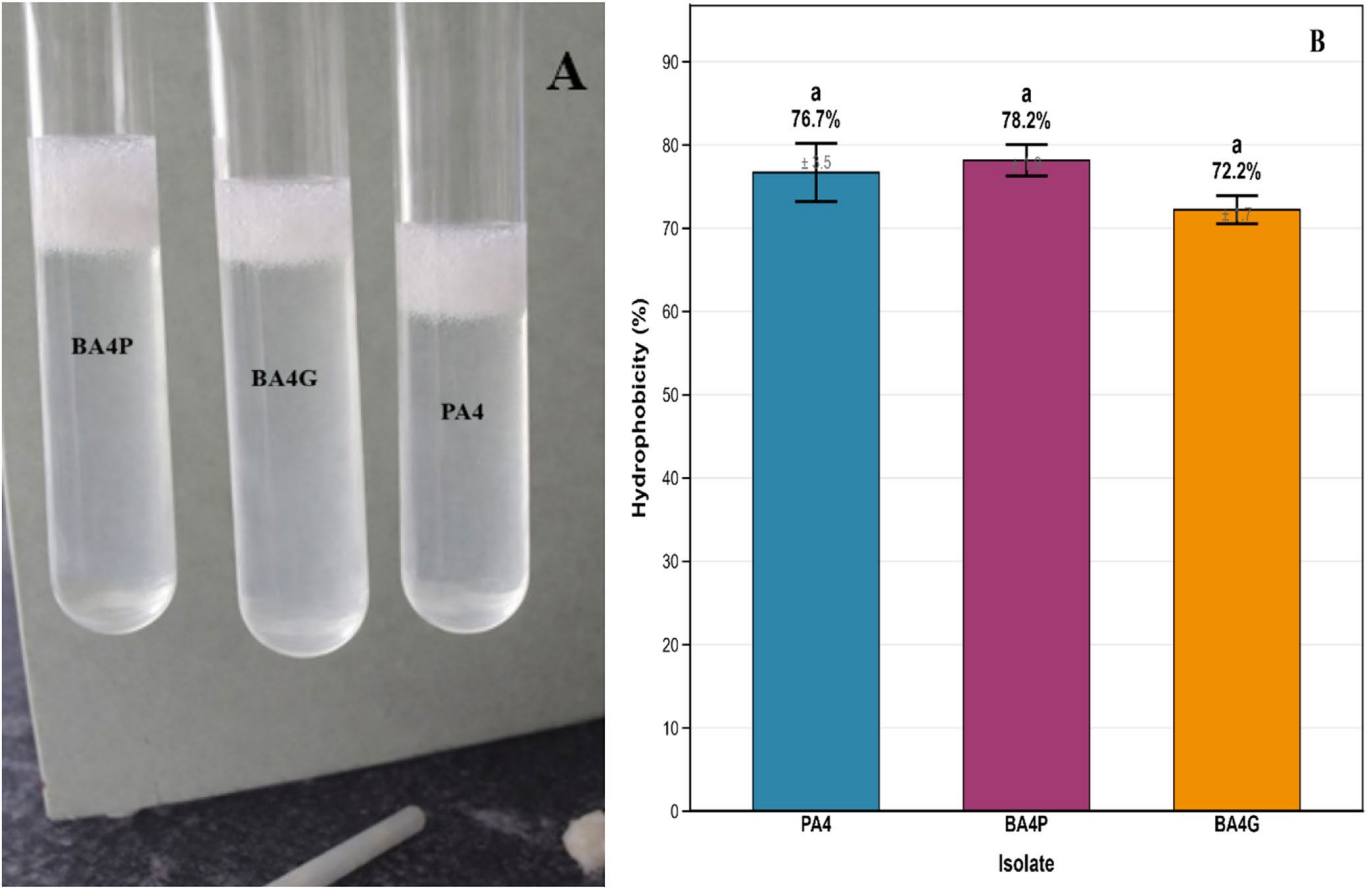
Cell surface hydrophobicity of probiotic candidates PA4, BA4P and BA4G evaluated using the microbial adhesion to hydrocarbons (xylene) assay. (A) Hydrophobicity observed after complete separation of the two phases. (B) Hydrophobicity expressed as percentage reduction in optical density relative to the initial cell suspension. Values represent mean ± SD. Different superscript letters indicate significant differences among isolates (one‐way ANOVA followed by Tukey's HSD test, *p* < 0.05).

### 3.5 Tolerance to Simulated Gastrointestinal Conditions

The tolerance of the shortlisted isolates to simulated gastrointestinal stress was evaluated under acid–pepsin and bile salts–pancreatin conditions over a 6‐h exposure period. Survival was expressed as percentage retention of OD_600_ relative to initial values. Under both stress conditions, OD_600_ retention generally declined with increasing exposure time; however, distinct isolate‐specific trends were observed. Notably, from the 4th hour onward, OD_600_ values for PA4 and BA4P stabilized and showed a slight recovery, indicating adaptive tolerance to prolonged stress, whereas BA4G continued to exhibit a progressive decline throughout the exposure period. The time‐dependent tolerance profiles are illustrated in Figure 5, with corresponding numerical values summarized in Table S3.

**FIGURE 5:**
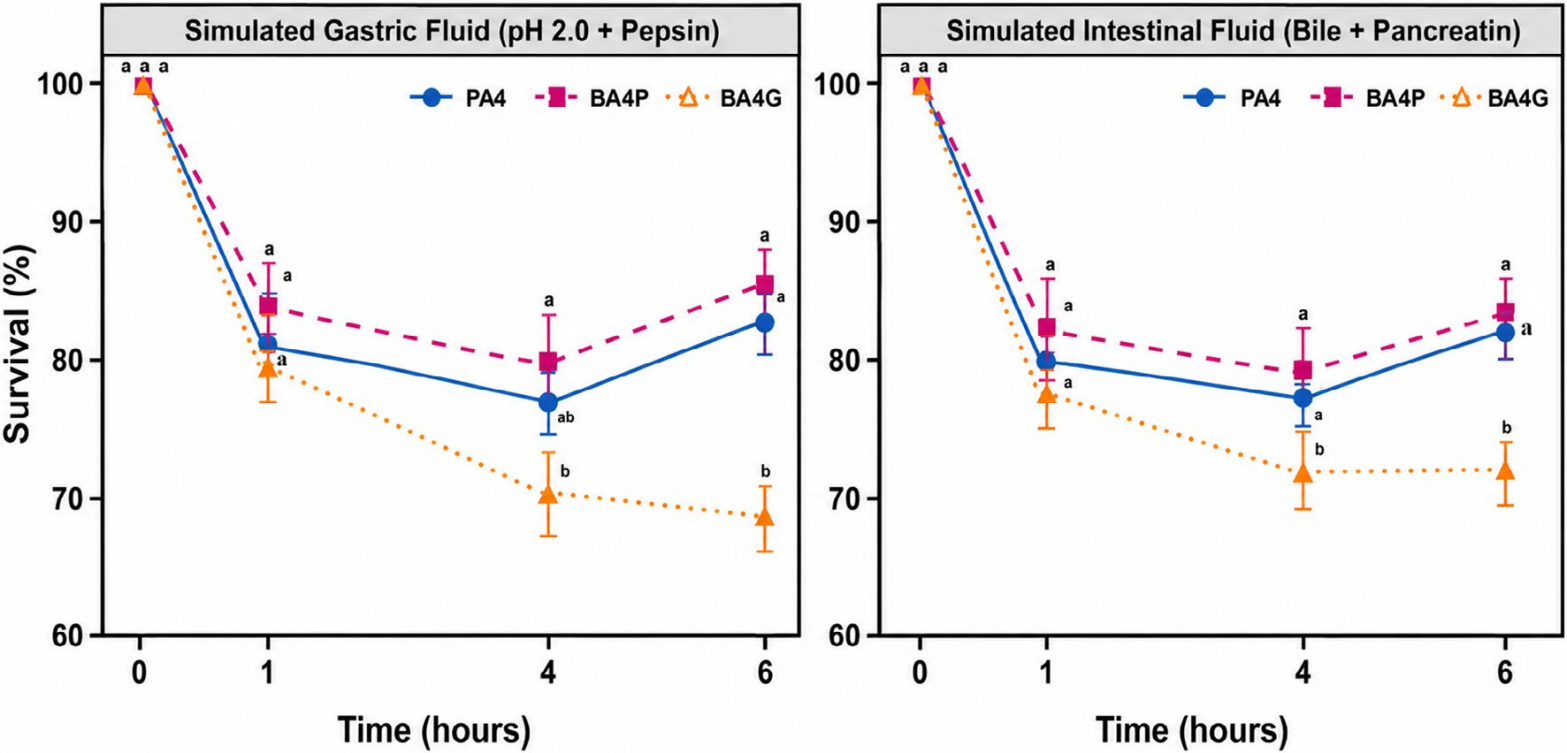
Tolerance of probiotic candidates PA4, BA4P and BA4G to simulated gastrointestinal conditions, acid–pepsin tolerance and bile–pancreatin tolerance evaluated over 0, 1, 4 and 6 h. Survival is expressed as percentage retention of optical density (OD_600_) relative to initial values. Data represent mean ± SD. Different superscript letters indicate significant differences among isolates at the same time point (one‐way ANOVA followed by Tukey's HSD, *p* < 0.05).

### 3.6 Growth Under Variable Salinity and Temperature

The growth performance of PA4, BA4P and BA4G was evaluated under different temperature (10°C–40°C) and salinity (0%–3% NaCl) conditions. Growth responses, expressed as relative OD_600_, varied among isolates depending on environmental conditions. Comparative growth profiles across temperature and salinity gradients are presented in Figure 6A,B respectively; while corresponding numerical values are provided in Table S4.

**FIGURE 6:**
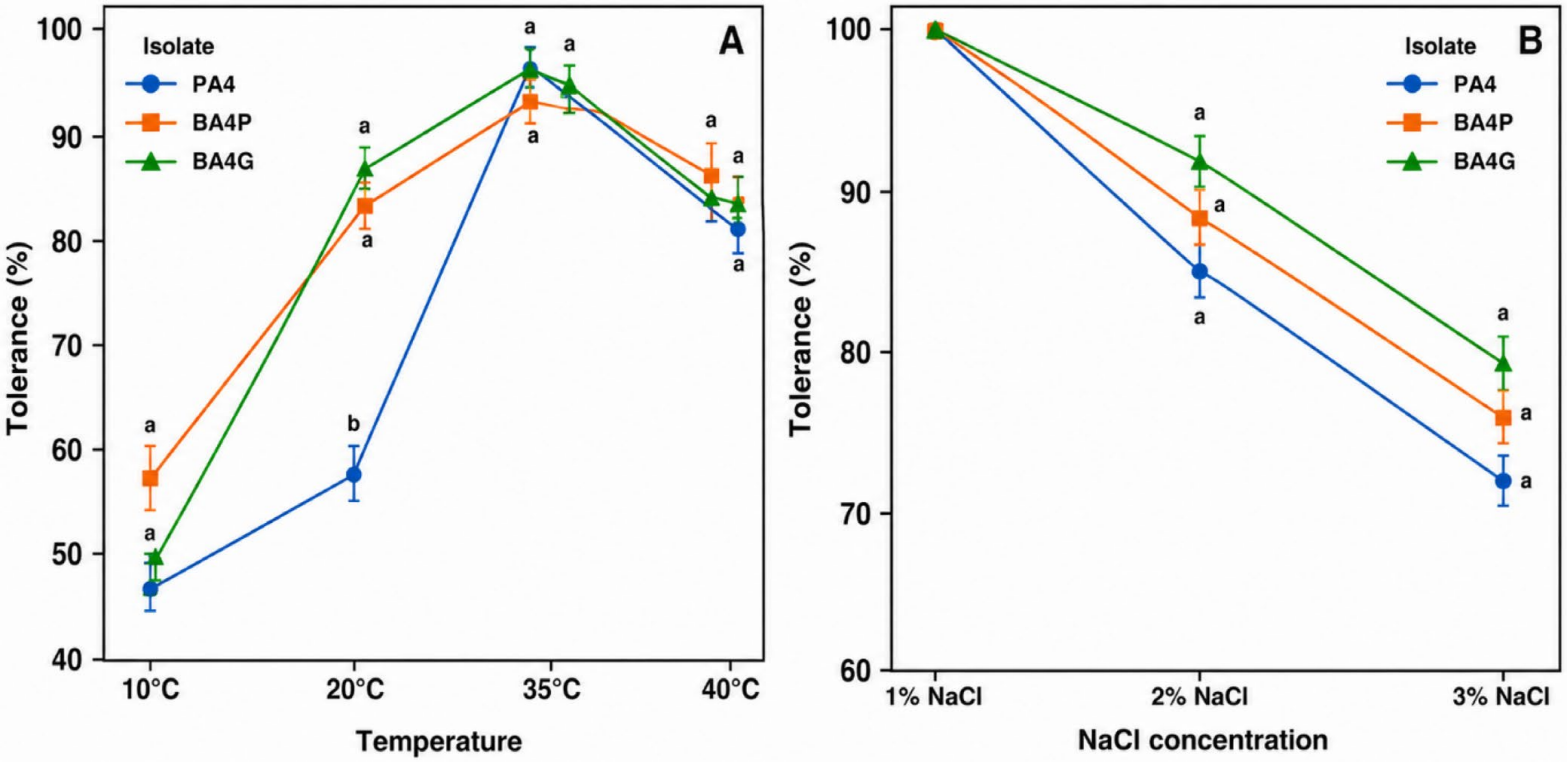
Growth response of probiotic candidates PA4, BA4P and BA4G under different environmental conditions. (A) Growth at 10°C, 20°C, 35°C and 40°C, and (B) growth at increasing salt levels (0%, 2% and 3% NaCl), expressed as percentage of optical density (OD_600_) relative to optimal conditions. The tests have been done in triplicate. Values represent mean ± SD. Different superscript letters within each condition indicate significant differences among isolates (one‐way ANOVA followed by Tukey's HSD, *p* < 0.05).

### 3.7 Enzymatic and Biochemical Activities of Shortlisted Isolates

The shortlisted isolates (PA4, BA4P and BA4G) were further characterized for key enzymatic and biochemical traits relevant to probiotic candidacy. As summarized in the enzymatic profiling results, PA4 showed positive reactions for protease, lipase, urease, phosphatase and amylase, while remaining negative for cellulase. BA4P exhibited protease, lipase, urease and cellulase activity, but did not show detectable amylase or phosphatase activity. BA4G showed urease, cellulase, phosphatase and amylase activity, but was negative for protease and lipase; all three isolates were negative for tryptophanase but positive for oxidase (Table 3). Tryptophanase (Indole) test results are presented in Figure 7A, urease activity in Figure 7B, lipase activity on tributyrin agar in Figure 7C, oxidase activity in Figure 7D amylase activity on starch agar in Figure 7E and protease activity in Figure 7F.

**TABLE 3:** Biochemical, enzymatic and phenotypic characteristics of the selected isolates.

Test	PA 4	BA4G	BA4P
Tryptophanase	−	−	−
Amylase	+	+	−
Urease	+	+	+
Protease	+	−	+
Cellulase	−	+	+
Oxidase	+	+	+
Phosphatase	+	+	−
Lipase	+	−	+
Gram	−	+	+
Endospores	−	+	+
Siderophores	+	+	+
Motility	+	+	+

**FIGURE 7:**
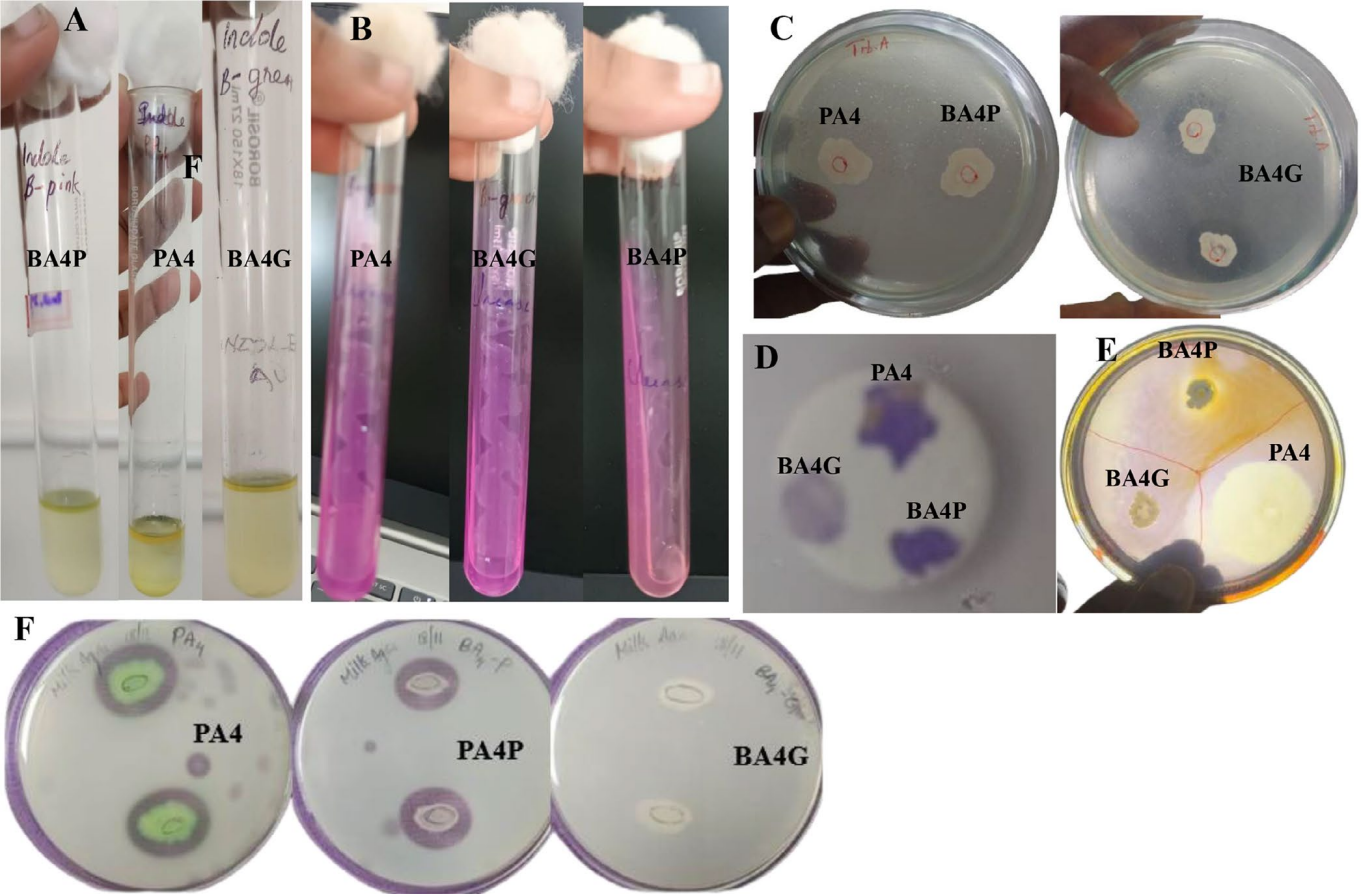
Some of the enzymatic and biochemical activities of selected isolates: Indole test (A), urease activity (B), lipase activity on tributyrin agar (C), oxidase activity (D), amylase activity on starch agar (E) and protease activity (F).

### 3.8 Other Characteristics of the Selected Candidates

Additional screening of PA4, BA4P and BA4G indicated that the isolates differed in Gram reaction and spore‐forming capacity. PA4 was Gram‐negative and non‐spore‐forming, whereas BA4P and BA4G were Gram‐positive and endospore‐forming. All three isolates produced siderophores on CAS agar and exhibited motility in motility assays. These core phenotypic characteristics are summarized alongside enzymatic profiles in Table 3.

### 3.9 Down‐Selection of Probiotic Candidates

Following completion of antimicrobial, safety, gastrointestinal tolerance, adhesion‐related and environmental robustness screening, PA4 and BA4P were selected for downstream molecular identification and further characterization. Although BA4G passed the safety assessment, it showed comparatively lower performance in functional screening assays and was therefore not advanced to molecular identification.

### 3.10 Molecular Identification of Selected Isolates

The taxonomic identity of the selected probiotic candidates PA4 and BA4P was determined based on 16S rRNA gene sequence analysis. Near full‐length 16S rRNA gene fragments were amplified using universal bacterial primers, and bidirectional sequencing was performed. Forward and reverse sequences were assembled to generate high‐quality consensus sequences for each isolate after trimming and quality control. BLAST analysis of the assembled 16S rRNA gene sequence of isolate PA4 revealed a high level of sequence similarity with several strains of *Pseudomonas aeruginosa* including strains corresponding to GenBank accession numbers HQ844513.1 and LC648441.1, with 99.93% sequence identity with a 100% query coverage. The assembled 16S rRNA gene sequence of isolate BA4P showed highest similarity to *Bacillus* sp., with 100% sequence identity and 100% query coverage to the reference sequence (GenBank accession number: FJ785481.1). The strain also showed a close (99.93% identity) similarity to several strains of *Bacillus flexus* (*Priestia flexa*) with a 100% query coverage. In the resulting phylogenetic tree, isolate PA4 clustered within the *Pseudomonas aeruginosa* clade (Figure 8A), while BA4P grouped with *Bacillus* sp. (Figure 8B). Hence PA4 has been identified as *Pseudomonas aeruginosa* PA4 (GenBank accession number: PX766316) and BA4P as *Bacillus* sp. BA4P (GenBank accession number: PX766366.2). Figure 8C shows the agarose gel electrophoresis image of the two selected probiotic candidates.

**FIGURE 8:**
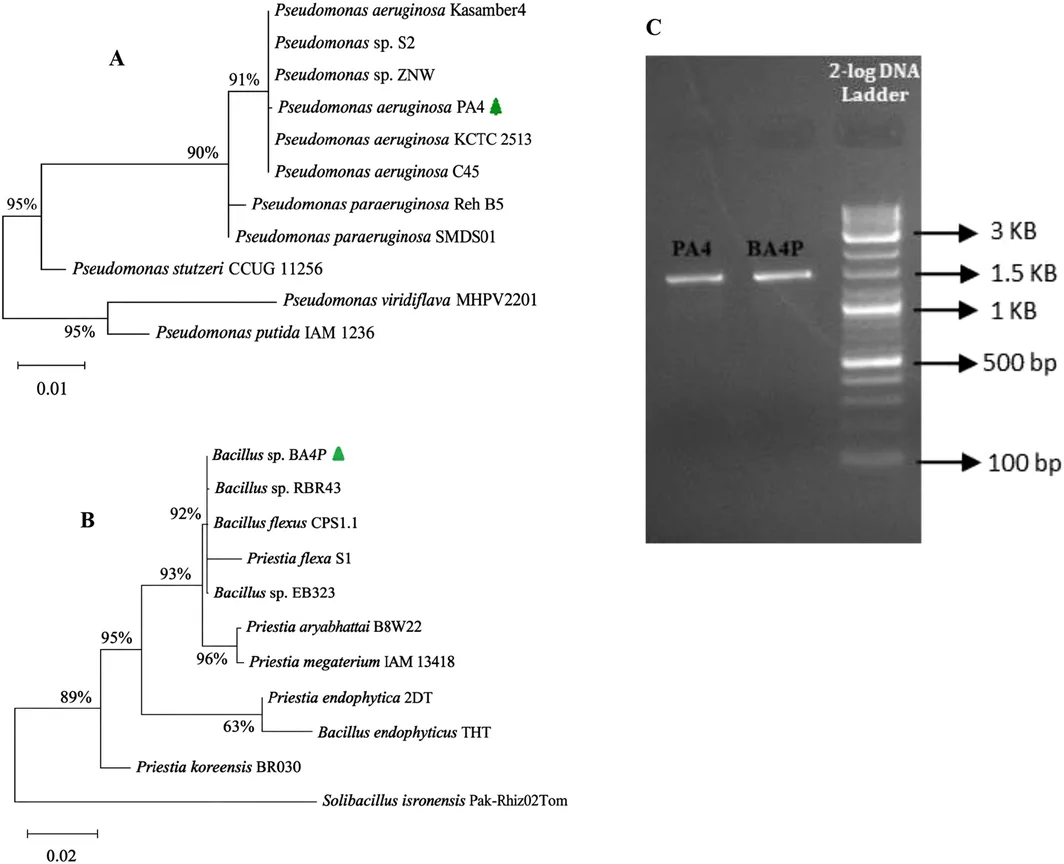
The phylogenetic trees of selected probiotic candidates BA4P (A) and PA4 (B) with percentage of site coverage and the PCR product quality with agarose gel electrophoresis (C).

## 4 Discussion

Probiotic selection in aquaculture requires integration of multiple functional traits, as no single parameter reliably predicts in vivo efficacy (Calcagnile et al. 2024; Rahayu et al. 2024). Accordingly, a stepwise screening framework (Hasan and Banerjee 2020) was applied to evaluate autochthonous gut bacteria from Red Tilapia across antimicrobial activity, safety, gastrointestinal tolerance, adhesion‐related traits, enzymatic capacity, environmental robustness and taxonomic identity. The marked reduction from 26 isolates to two candidates (PA4 and BA4P) reflects the inherent selectivity of probiotic screening, where only a small fraction of gut microbiota exhibits consistent probiotic‐relevant traits (Mazanko et al. 2025; Rwezawula et al. 2025; Sabino et al. 2025). The exclusion of BA4G at later stages, despite early positive traits, highlights that functional robustness under physiologically relevant stress (e.g., gastrointestinal simulation) is a decisive criterion. This supports previous assertions that probiotic selection should rely on quantitative, multi‐trait performance thresholds rather than binary screening outcomes (Binda et al. 2020; Pramanik et al. 2023; Yadav and Shukla 2017).

Antagonism against fish pathogens is widely used as an initial filter due to the high disease pressure in intensive aquaculture systems (Bhandary et al. 2021; Binda et al. 2020; Sachdeva et al. 2025). In line with previous studies, antimicrobial activity was restricted to a minority of isolates, indicating that pathogen inhibition is not a universal trait within gut microbiota (Chauhan and Singh 2019a; Govindaraj et al. 2021; Yao et al. 2024). The broad‐spectrum inhibition exhibited by PA4 and BA4P suggests competitive advantages mediated by mechanisms such as nutrient competition, antimicrobial metabolite production or niche exclusion (Hou et al. 2025; Sharma and Lee 2025; Wang et al. 2025). However, consistent with current recommendations, antimicrobial activity alone was not considered sufficient for probiotic selection, but rather a gatekeeping criterion ensuring that only isolates with pathogen suppression potential proceed to further evaluation.

Safety remains a primary requirement for probiotic application in food‐producing animals (Lahtinen et al. 2009; Lin et al. 2025; Sanders et al. 2010). The absence of haemolytic activity and the broad antibiotic susceptibility profiles observed here are consistent with accepted safety benchmarks for candidate probiotics (Keter et al. 2022; Muñoz‐Atienza et al. 2013). Importantly, in vivo exposure did not induce mortality or physiological disturbance, indicating host tolerability under systemic challenge conditions (Calcagnile et al. 2024). The survival of the control group remained within the range observed for the probiotic‐treated groups throughout the 14‐day observation period, although it was generally lower than that of the treated groups. Despite this apparent difference, no significant differences were detected among groups and the overall survival patterns provide a reference for evaluating the survival response of fish administered the candidate isolates. This conservative approach strengthens safety inference by testing beyond oral exposure scenarios (Calcagnile et al. 2024; Simón et al. 2021). Collectively, these results support the view that early integration of in vitro and in vivo safety assessments reduces downstream application risks and is essential for responsible probiotic development.

Survival under gastrointestinal stress is a prerequisite for probiotic functionality, as strains must remain viable during transit to exert effects in the intestine (Fernández de Palencia et al. 2008; Goderska and Agudo Pena 2024; Stasiak‐Różańska et al. 2021). The sustained tolerance of PA4 and BA4P under acid–pepsin and bile–pancreatin conditions, particularly at extended exposure times, indicates functional resilience rather than transient survival, a distinction increasingly emphasized in probiotic screening (Chauhan and Singh 2019b; Yao et al. 2024). Higher cell surface hydrophobicity further suggests enhanced interaction with epithelial surfaces, which may facilitate persistence within the gut environment (Farid et al. 2021). Although hydrophobicity is only an indirect proxy for adhesion, its consistent application as a screening parameter supports its utility when interpreted alongside stress tolerance data (Mirzabekyan et al. 2023; Sadeghi, Ebrahimi, et al. 2022; Sadeghi, Panahi, et al. 2022). Together, these traits indicate that colonization potential emerges from the combined effect of stress resistance and surface interaction capacity.

Extracellular enzyme production is a key functional trait linking probiotics to improved host nutrition (Maas, Deng, et al. 2021; Maas, Verdegem, et al. 2021; Srirengaraj et al. 2023). The enzymatic profiles observed here suggest complementary functional roles, with PA4 exhibiting a broader enzymatic spectrum and BA4P demonstrating strong proteolytic and lipolytic capacities. Such activities are known to enhance feed digestibility and nutrient assimilation in tilapia (Hlordzi et al. 2020; Wang et al. 2021). The absence of tryptophanase activity is also noteworthy, as indole production has been associated with undesirable metabolic effects (Deng et al. 2022; Tennoune et al. 2022). While enzymatic activity alone does not guarantee in vivo efficacy, it provides mechanistic support for potential probiotic effects and reinforces findings from antimicrobial and tolerance assays (Guarnieri et al. 2025; Saini et al. 2026). The observed differences in enzymatic profiles among the selected isolates likely reflect species‐specific metabolic capabilities and ecological adaptations. *Pseudomonas* spp. are recognized for their broad metabolic versatility and capacity to produce diverse extracellular enzymes (Hoque et al. 2019), whereas *Bacillus* spp. are well known for secreting hydrolytic enzymes such as proteases and lipases (El‐Son et al. 2022; Midhun et al. 2019). These complementary enzymatic traits may contribute to different mechanisms by which host‐derived probiotics enhance nutrient digestion and utilization in fish. The functional relevance of these in vitro characteristics has subsequently been supported by an independent feeding trial, in which dietary supplementation with PA4 and BA4P significantly improved growth performance, gut health, immune responses and disease resistance in red tilapia (Ntakirutimana et al. 2026). These findings suggest that the screening framework adopted in the present study successfully identified probiotic candidates with demonstrable in vivo efficacy.

Environmental tolerance is critical for probiotic performance under practical aquaculture conditions, where temperature and salinity fluctuations are common (Lorgen‐Ritchie et al. 2023; Mugwanya et al. 2022). The stable growth of PA4 and BA4P across temperature and salinity gradients indicates adaptability that may enhance persistence in production systems. Such robustness has been linked to improved probiotic reliability in previous studies (Ganguly 2019; Ganguly et al. 2019; Yao et al. 2024). Therefore, environmental tolerance should be considered not only a survival trait but also a determinant of functional consistency under farm conditions. Although the multi‐criteria screening strategy enabled systematic selection of promising probiotic candidates, several limitations should be acknowledged. The study focused on culturable intestinal bacteria recovered under laboratory conditions; therefore, potentially beneficial unculturable or low‐abundance microorganisms may not have been represented. In addition, candidate selection relied primarily on phenotypic characterization, and further molecular analyses of virulence‐associated genes and transferable antibiotic resistance determinants would strengthen the safety assessment before practical application.

The identification of PA4 as *Pseudomonas aeruginosa* and BA4P as *Bacillus* sp. provides a biological framework for interpreting their functional traits. Both genera are commonly associated with fish microbiota and are adapted to dynamic aquatic environments (James et al. 2021; Korkea‐aho et al. 2011; Preetha et al. 2010, 2015; Tachibana et al. 2021), which likely underpins their observed tolerance to gastrointestinal and environmental stress. The strong antimicrobial activity of PA4 can be linked to genus‐specific mechanisms, including siderophore production and secondary metabolite synthesis of *Pseudomonas* species (Giri et al. 2020; Kharabsheh et al. 2017; Ntakirutimana et al. 2025; Preetha et al. 2010, 2015). Siderophore‐mediated iron sequestration, observed in this study, is a well‐established mechanism limiting pathogen proliferation in aquatic systems (Devi et al. 2022; Si et al. 2024). Similar inhibitory profiles have been reported for fish‐derived *Pseudomonas* strains, supporting the ecological relevance of these mechanisms (Hoque et al. 2019; Hoque and Abraham 2021; Vinoj et al. 2013). The identification of *P. aeruginosa* necessitates careful consideration due to its association with opportunistic pathogenicity. However, previous studies have demonstrated that environmental strains can exhibit probiotic‐like traits when virulence determinants are absent (Giri et al. 2012, 2014; Hoque et al. 2019; Hoque and Abraham 2021; Ntakirutimana et al. 2025). The non‐haemolytic phenotype, antibiotic susceptibility and in vivo safety observed here suggest that PA4 represents a non‐virulent strain, although further molecular characterization would be required for application.

The classification of BA4P within *Bacillus* is consistent with its functional robustness. Endospore formation provides resistance to environmental stress and processing conditions, supporting its persistence and applicability in aquaculture systems (Galagarza et al. 2018; Konieczka et al. 2023; Nayak 2021). Its enzymatic and antimicrobial traits align with well‐established probiotic mechanisms in *Bacillus*, including enzyme secretion and production of lipopeptides and bacteriocins (Dias et al. 2022; Mukherjee and Ghosh 2016; Pereira et al. 2022; Yu et al. 2025). The taxonomic identities of PA4 and BA4P therefore reflect complementary functional strategies: competitive antimicrobial activity (*Pseudomonas*) and environmental resilience with nutritional contribution (*Bacillus*). Similar multi‐strain approaches have been shown to enhance probiotic efficacy compared with single‐strain applications (Abd El‐Rhman et al. 2009; Giri et al. 2014; Vinoj et al. 2013). The taxonomic identification enabled functional interpretation rather than merely classification, reinforcing that probiotic performance is best understood at the intersection of taxonomy, phenotype and safety. Collectively, these findings illustrate how taxon‐specific metabolic and structural traits iron‐scavenging and secondary metabolite production in *Pseudomonas*, and sporulation‐linked stress resistance in *Bacillus* mediate microbial persistence and competitive interactions within the host gut environment. This reinforces the view that microbial functional diversity in aquatic hosts is shaped by the interplay between taxonomic identity and environmental adaptation, extending beyond their immediate value as probiotic candidates.

## 5 Conclusion

This study applied a systematic, multi‐criteria screening strategy to identify autochthonous probiotic candidates from the intestinal microbiota of healthy red tilapia. From an initial pool of 26 isolates, progressive evaluation of antimicrobial activity, safety, gastrointestinal tolerance, adhesion‐related traits, enzymatic potential, environmental robustness and molecular identification enabled the selection of two promising candidates, *Pseudomonas aeruginosa* PA4 and *Bacillus* sp. BA4P. Both strains exhibited broad‐spectrum antagonistic activity against major fish pathogens, favourable safety profiles supported by non‐haemolytic behaviour, antibiotic susceptibility and in vivo safety evaluation, together with functional characteristics consistent with probiotic potential. These findings demonstrate that a comprehensive, phenotype‐based screening strategy is effective for identifying host‐associated probiotic candidates for aquaculture and provides a foundation for the development of species‐specific probiotics for red tilapia. Nevertheless, further molecular characterization, including evaluation of virulence‐associated and antibiotic resistance genes, remains necessary to comprehensively establish the biosafety of the selected strains prior to their large‐scale application in aquaculture. More broadly, this work contributes to understanding how gut‐associated microorganisms combine functional traits (enzymatic activity, antimicrobial metabolite production) with environmental resilience to persist and compete within host‐associated niches, informing the wider study of host–microbe–environment interactions in aquatic systems.

## Author Contributions

**Remy Ntakirutimana:** conceptualization, writing – original draft, methodology, writing – review and editing, data curation, software, formal analysis, validation. **Mariya Jacob Ashin:** validation, data curation, visualization. **Aishath Ali Farhath:** data curation, validation, visualization, writing – review and editing. **Anatole Bigirimana:** methodology, validation, visualization, writing – review and editing. **N. Regina Hershey:** visualization, formal analysis, validation, writing – review and editing. **Megha Lovejan:** visualization, data curation, writing – review and editing. **Paul Mwangi:** formal analysis, writing – review and editing, validation. **M. P. Jijina:** data curation, validation, writing – review and editing. **Fazeena Fariq:** visualization, writing – review and editing, validation, formal analysis. **C. Varsha:** data curation, validation, visualization. **K. M. Mujeeb Rahiman:** conceptualization, supervision, methodology, validation, visualization, writing – review and editing.

## Funding

The authors have nothing to report.

## Ethics Statement

All experimental procedures involving fish were reviewed and approved by the Institutional Animal Ethics Committee (IAEC) of Cochin University of Science and Technology (Protocol No. 363/GO/Re/S/01/CCSEA/115, meeting date: 19.12.2024). The study was conducted in accordance with relevant national guidelines for the care and use of laboratory animals.

## Conflicts of Interest

The authors declare no conflicts of interest.

## Supporting information


**Table S1:** Morphometric characteristics of colonies isolated from red tilapia gut.
**Table S2:** Screening of the antagonism activity of candidates with pathogens.
**Table S3:** Tolerance to simulated gastrointestinal conditions (survival %).
**Table S4:** Tolerance to variable salinity and temperature (survival %).

## Data Availability

The data that support the findings of this study are openly available in Zenodo repository at https://doi.org/10.5281/zenodo.19495555, reference number 19495555.

## References

[emi470438-bib-0001] Abd El‐Rhman, A. M. , Y. A. E. Khattab , and A. M. E. Shalaby . 2009. “ *Micrococcus luteus* and *Pseudomonas* Species as Probiotics for Promoting the Growth Performance and Health of Nile Tilapia, *Oreochromis niloticus* .” Fish and Shellfish Immunology 27, no. 2: 175–180. 10.1016/j.fsi.2009.03.020. 19361560

[emi470438-bib-0002] Abdel‐Tawwab, M. , M. A. A. Mousa , and M. A. Mohammed . 2010. “Use of Live Baker's Yeast, *Saccharomyces cerevisiae* , in Practical Diet to Enhance the Growth Performance of Galilee Tilapia, *Sarotherodon galilaeus* (L.), and Its Resistance to Environmental Copper Toxicity.” Journal of the World Aquaculture Society 41, no. Suppl. 2: 214–223. 10.1111/j.1749-7345.2010.00361.x.

[emi470438-bib-0003] Abdul Manan, M. 2025. “The Role of Probiotics in Personalized Therapeutics: Advances in Gut Microbe‐Driven Interventions.” Microbe 8: 100497. 10.1016/j.microb.2025.100497.

[emi470438-bib-0110] Amal, M. N. A. , C. B. Koh , M. Nurliyana , et al. 2018. “A Case of Natural Co‐Infection of Tilapia Lake Virus and Aeromonas Veronii in a Malaysian Red Hybrid Tilapia (Oreochromis Niloticus × O. Mossambicus) Farm Experiencing High Mortality.” Aquaculture 485: 12–16. 10.1016/j.aquaculture.2017.11.019.

[emi470438-bib-0004] Banerjee, G. , and A. K. Ray . 2017. “The Advancement of Probiotics Research and Its Application in Fish Farming Industries.” Research in Veterinary Science 115: 66–77. 10.1016/j.rvsc.2017.01.016. 28157611

[emi470438-bib-0005] Beck, B. R. , D. Kim , J. Jeon , et al. 2015. “The Effects of Combined Dietary Probiotics *Lactococcus lactis* BFE920 and *Lactobacillus plantarum* FGL0001 on Innate Immunity and Disease Resistance in Olive Flounder ( *Paralichthys olivaceus* ).” Fish and Shellfish Immunology 42, no. 1: 177–183. 10.1016/j.fsi.2014.10.035. 25449382

[emi470438-bib-0006] Bhandary, T. , L. Riyaz Ali , and K. A. Paari . 2021. “Probiotic Properties of *Bacillus subtilis* Isolated From Dried Anchovies ( *Stolephorus indicus* ) and Evaluating Its Antimicrobial, Antibiofilm and Growth‐Enhancing Potential in *Danio rerio* .” Journal of Animal Health and Production 9, no. 3: 205–212. 10.17582/JOURNAL.JAHP/2021/9.3.205.212.

[emi470438-bib-0007] Binda, S. , C. Hill , E. Johansen , et al. 2020. “Criteria to Qualify Microorganisms as “Probiotic” in Foods and Dietary Supplements.” Frontiers in Microbiology 11: 1662. 10.3389/fmicb.2020.01662. PMC7394020 32793153

[emi470438-bib-0008] Bona, A. M. , L. S. Passos , G. C. Coppo , et al. 2024. “Enhancing Anesthesia and Minimizing Toxicity: Evaluation of Clove, Cinnamon, and Tea Tree Essential Oils in Nile Tilapia ( *Oreochromis niloticus* ).” Aquaculture International 32: 6691–6706. 10.1007/s10499-024-01484-6.

[emi470438-bib-0009] Calcagnile, M. , S. M. Tredici , and P. Alifano . 2024. “A Comprehensive Review on Probiotics and Their Use in Aquaculture: Biological Control, Efficacy, and Safety Through the Genomics and Wet Methods.” Heliyon 10, no. 24: e40892. 10.1016/j.heliyon.2024.e40892. PMC11681891 39735631

[emi470438-bib-0010] Chauhan, A. , and R. Singh . 2019a. “Isolation and Evaluation of Putative Probiotic Strains From Different Teleost to Prevent *Pseudomonas aeruginosa* Infection in *Cyprinus carpio* .” Aquaculture Research 50, no. 12: 3616–3627. 10.1111/are.14318.

[emi470438-bib-0011] Chauhan, A. , and R. Singh . 2019b. “Probiotics in Aquaculture: A Promising Emerging Alternative Approach.” Symbiosis 77, no. 2: 99–113. 10.1007/s13199-018-0580-1.

[emi470438-bib-0012] Coulibaly, W. H. , N. R. Kouadio , F. Camara , C. Diguță , and F. Matei . 2023. “Functional Properties of Lactic Acid Bacteria Isolated From Tilapia ( *Oreochromis niloticus* ) in Ivory Coast.” BMC Microbiology 23, no. 1: 152. 10.1186/s12866-023-02899-6. PMC10210322 37231432

[emi470438-bib-0013] Dawood, M. A. O. 2021. “Nutritional Immunity of Fish Intestines: Important Insights for Sustainable Aquaculture.” Reviews in Aquaculture 13, no. 1: 642–663. 10.1111/raq.12492.

[emi470438-bib-0014] Deng, J.‐J. , D. Deng , Z.‐L. Wang , et al. 2022. “Indole Metabolism Mechanisms in a New, Efficient Indole‐Degrading Facultative Anaerobe Isolate *Enterococcus hirae* GDIAS‐5.” Journal of Hazardous Materials 434: 128890. 10.1016/j.jhazmat.2022.128890. 35452978

[emi470438-bib-0015] Devi, A. A. , M. I. R. Khan , T. G. Choudhury , and D. Kamilya . 2022. “In Vitro Assessment of Probiotic Potential of an Autochthonous Bacterial Isolate, *Pseudomonas mosselii* COFCAU_PMP5.” Microbiology 91, no. 2: 207–214. 10.1134/s0026261722020047.

[emi470438-bib-0016] Dias, J. A. R. , L. L. Alves , F. A. L. Barros , et al. 2022. “Comparative Effects of Using a Single Strain Probiotic and Multi‐Strain Probiotic on the Productive Performance and Disease Resistance in *Oreochromis niloticus* .” Aquaculture 550: 737855. 10.1016/j.aquaculture.2021.737855.

[emi470438-bib-0111] Done, H. Y. , A. K. Venkatesan , and R. U. Halden . 2015. “Does the Recent Growth of Aquaculture Create Antibiotic Resistance Threats Different From Those Associated With Land Animal Production in Agriculture?” AAPS Journal 17, no. 3: 513–524. 10.1208/s12248-015-9722-z. PMC4406955 25700799

[emi470438-bib-0017] El‐Son, M. A. M. , G. E. Elshopakey , S. Rezk , E. A. A. Eldessouki , and S. Elbahnaswy . 2022. “Dietary Mixed *Bacillus* Strains Promoted the Growth Indices, Enzymatic Profile, Intestinal Immunity, and Liver and Intestinal Histomorphology of Nile Tilapia, *Oreochromis niloticus* .” Aquaculture Reports 27: 101385. 10.1016/j.aqrep.2022.101385.

[emi470438-bib-0018] Ertuǧrul, S. , G. Dönmez , and S. Takaç . 2007. “Isolation of Lipase Producing *Bacillus* sp. From Olive Mill Wastewater and Improving Its Enzyme Activity.” Journal of Hazardous Materials 149, no. 3: 720–724. 10.1016/j.jhazmat.2007.04.034. 17532131

[emi470438-bib-0019] FAO . 2022. The State of World Fisheries and Aquaculture 2022. FAO. 10.4060/cc0461en.

[emi470438-bib-0020] Farid, W. , T. Masud , A. Sohail , et al. 2021. “Gastrointestinal Transit Tolerance, Cell Surface Hydrophobicity, and Functional Attributes of *Lactobacillus acidophilus* Strains Isolated From Indigenous Dahi.” Food Science & Nutrition 9, no. 9: 5092–5102. 10.1002/fsn3.2468. PMC8441270 34532018

[emi470438-bib-0021] Fernández de Palencia, P. , P. López , A. L. Corbí , C. Peláez , and T. Requena . 2008. “Probiotic Strains: Survival Under Simulated Gastrointestinal Conditions, In Vitro Adhesion to Caco‐2 Cells and Effect on Cytokine Secretion.” European Food Research and Technology 227, no. 5: 1475–1484. 10.1007/s00217-008-0870-6.

[emi470438-bib-0022] Galagarza, O. A. , S. A. Smith , D. J. Drahos , J. D. Eifert , R. C. Williams , and D. D. Kuhn . 2018. “Modulation of Innate Immunity in Nile Tilapia ( *Oreochromis niloticus* ) by Dietary Supplementation of *Bacillus subtilis* Endospores.” Fish and Shellfish Immunology 83: 171–179. 10.1016/j.fsi.2018.08.062. 30176332

[emi470438-bib-0023] Ganguly, A. 2019. “Assessment of Growth and Nutrient Status of Indigenous Fresh Water Catfish ( *Clarias batrachus* Linn.) in Relation to the Application of Newly Isolated Probiotics.”

[emi470438-bib-0024] Ganguly, A. , A. Banerjee , A. Mandal , M. A. Khan , and P. K. D. Mohapatra . 2019. “Isolation and Characterization of Bacteria From the Intestine of *Clarias batrachus* for Probiotic Organism.” Proceedings of the Zoological Society 72, no. 4: 411–419. 10.1007/s12595-018-0283-x.

[emi470438-bib-0025] Gewaily, M. S. , M. Shukry , M. F. Abdel‐Kader , et al. 2021. “Dietary *Lactobacillus plantarum* Relieves Nile Tilapia ( *Oreochromis niloticus* ) Juvenile From Oxidative Stress, Immunosuppression, and Inflammation Induced by Deltamethrin and *Aeromonas hydrophila* .” Frontiers in Marine Science 8: 621558. 10.3389/fmars.2021.621558.

[emi470438-bib-0026] Giri, S. S. , J. W. Jun , S. Yun , et al. 2020. “Effects of Dietary Heat‐Killed *Pseudomonas aeruginosa* Strain VSG2 on Immune Functions, Antioxidant Efficacy, and Disease Resistance in *Cyprinus carpio* .” Aquaculture 514: 734489. 10.1016/j.aquaculture.2019.734489.

[emi470438-bib-0027] Giri, S. S. , S. S. Sen , and V. Sukumaran . 2012. “Effects of Dietary Supplementation of Potential Probiotic *Pseudomonas aeruginosa* VSG‐2 on the Innate Immunity and Disease Resistance of Tropical Freshwater Fish, *Labeo rohita* .” Fish and Shellfish Immunology 32, no. 6: 1135–1140. 10.1016/j.fsi.2012.03.019. 22465361

[emi470438-bib-0028] Giri, S. S. , V. Sukumaran , S. S. Sen , and P. K. Jena . 2014. “Effects of Dietary Supplementation of Potential Probiotic *Bacillus subtilis* VSG1 Singularly or in Combination With *Lactobacillus plantarum* VSG3 or/and *Pseudomonas aeruginosa* VSG2 on the Growth, Immunity and Disease Resistance of *Labeo rohita* .” Aquaculture Nutrition 20, no. 2: 163–171. 10.1111/anu.12062.

[emi470438-bib-0029] Goderska, K. , and S. Agudo Pena . 2024. “An In Vitro Gastrointestinal Model to Evaluate the Tolerance of Encapsulated *Lactobacillus* and *Lactococcus* Strains With Synbiotic Containing Lactobionic Acid via Lyophilization Technique to Harsh Gastric Conditions During Storage Time.” European Journal of Pharmaceutics and Biopharmaceutics 197: 114147. 10.1016/j.ejpb.2023.11.012. 37967725

[emi470438-bib-0030] Govindaraj, K. , V. Samayanpaulraj , V. Narayanadoss , and R. Uthandakalaipandian . 2021. “Isolation of Lactic Acid Bacteria From Intestine of Freshwater Fishes and Elucidation of Probiotic Potential for Aquaculture Application.” Probiotics and Antimicrobial Proteins 13, no. 6: 1598–1610. 10.1007/s12602-021-09811-6. 34164781

[emi470438-bib-0031] Guarnieri, A. , M. Falcone , N. Brancazio , et al. 2025. “In Vivo Functional Screening on Innate Immunity of Lactic Acid Bacteria in *Galleria mellonella* Preclinical Model: Comparative Analysis of *Lactiplantibacillus plantarum* and *Lentilactobacillus kefiri* .” Frontiers in Cellular and Infection Microbiology 15: 1681687. 10.3389/fcimb.2025.1681687. PMC12549655 41141596

[emi470438-bib-0032] Guimarães, M. C. , I. M. Cerezo , M. F. Fernandez‐Alarcon , et al. 2022. “Oral Administration of Probiotics (*Bacillus subtilis* and *Lactobacillus plantarum* ) in Nile Tilapia ( *Oreochromis niloticus* ) Vaccinated and Challenged With *Streptococcus agalactiae* .” Fishes 7, no. 4: 211. 10.3390/fishes7040211.

[emi470438-bib-0033] Hasan, K. N. , and G. Banerjee . 2020. “Recent Studies on Probiotics as Beneficial Mediator in Aquaculture: A Review.” Journal of Basic and Applied Zoology 81, no. 1: 53. 10.1186/s41936-020-00190-y.

[emi470438-bib-0034] Hlordzi, V. , F. K. A. Kuebutornye , G. Afriyie , et al. 2020. “The Use of *Bacillus* Species in Maintenance of Water Quality in Aquaculture: A Review.” Aquaculture Reports 18: 100503. 10.1016/j.aqrep.2020.100503.

[emi470438-bib-0035] Hoque, F. , and T. J. Abraham . 2021. “Adhesion and Colonization of Potential Probiont *Pseudomonas aeruginosa* FARP72 in the Intestine of Yellowtail Catfish, *Pangasius pangasius* .” Archives of Microbiology 203, no. 5: 2711–2717. 10.1007/s00203-021-02188-8. 33585990

[emi470438-bib-0036] Hoque, F. , T. Jawahar Abraham , T. S. Nagesh , and D. Kamilya . 2019. “ *Pseudomonas aeruginosa* FARP72 Offers Protection Against *Aeromonas hydrophila* Infection in *Labeo rohita* .” Probiotics and Antimicrobial Proteins 11, no. 3: 973–980. 10.1007/s12602-018-9456-1. 30112591

[emi470438-bib-0037] Hou, Y. , Y. Duan , G. Wu , et al. 2025. “Antibacterial Activity, Probiotic Potential, and Biocontrol Efficacy of Two Lactic Acid Bacteria Against *Penicillium expansum* on Fresh Grapes.” Food 14, no. 3: 493. 10.3390/foods14030493. PMC11816955 39942086

[emi470438-bib-0038] Hussey, A. M. , and A. Zayaitz . 2007. Endospore Stain Protocol. Vol. 11. American Society for Microbiology. http://www.hpa.

[emi470438-bib-0039] Jain, A. , R. Jain , and S. Jain . 2020. Motility Testing—Hanging Drop Method and Stab, 121–122. Humana. 10.1007/978-1-4939-9861-6_34.

[emi470438-bib-0040] James, G. , B. C. Das , S. Jose , and R. K. Rejish . 2021. “ *Bacillus* as an Aquaculture Friendly Microbe.” Aquaculture International 29, no. 1: 323–353. 10.1007/s10499-020-00630-0.

[emi470438-bib-0041] Jang, W. J. , K. B. Lee , M. H. Jeon , et al. 2023. “Characteristics and Biological Control Functions of *Bacillus* sp. PM8313 as a Host‐Associated Probiotic in Red Sea Bream ( *Pagrus major* ) Aquaculture.” Animal Nutrition 12: 20–31. 10.1016/j.aninu.2022.08.011. PMC9641168 36381063

[emi470438-bib-0042] Kasana, R. C. , R. Salwan , H. Dhar , S. Dutt , and A. Gulati . 2008. “A Rapid and Easy Method for the Detection of Microbial Cellulases on Agar Plates Using Gram's Iodine.” Current Microbiology 57, no. 5: 503–507. 10.1007/s00284-008-9276-8. 18810533

[emi470438-bib-0043] Keter, M. T. , N. M. El Halfawy , and M. Y. El‐Naggar . 2022. “Incidence of Virulence Determinants and Antibiotic Resistance in Lactic Acid Bacteria Isolated From Food Products.” Future Microbiology 17, no. 5: 325–337. 10.2217/fmb-2021-0053. 35172602

[emi470438-bib-0044] Kharabsheh, H. A. , S. Han , S. Allen , and S. L. Chao . 2017. “Metabolism of Chlorpyrifos by *Pseudomonas aeruginosa* Increases Toxicity in Adult Zebrafish ( *Danio rerio* ).” International Biodeterioration & Biodegradation 121: 114–121. 10.1016/j.ibiod.2017.03.024.

[emi470438-bib-0045] Konieczka, P. , K. Ferenc , J. N. Jørgensen , et al. 2023. “Feeding *Bacillus*‐Based Probiotics to Gestating and Lactating Sows Is an Efficient Method for Improving Immunity, Gut Functional Status and Biofilm Formation by Probiotic Bacteria in Piglets at Weaning.” Animal Nutrition 13: 361–372. 10.1016/j.aninu.2023.03.003. PMC10300407 37388456

[emi470438-bib-0046] Korkea‐aho, T. L. , J. Heikkinen , K. D. Thompson , A. von Wright , and B. Austin . 2011. “ *Pseudomonas* sp. M174 Inhibits the Fish Pathogen *Flavobacterium psychrophilum* .” Journal of Applied Microbiology 111, no. 2: 266–277. 10.1111/j.1365-2672.2011.05044.x. 21554504

[emi470438-bib-0047] Lahtinen, S. J. , R. J. Boyle , A. Margolles , R. Frias , and M. Gueimonde . 2009. “Safety Assessment of Probiotics.” In Prebiotics and Probiotics Science and Technology, 1193–1235. Springer. 10.1007/978-0-387-79058-9_31.

[emi470438-bib-0048] Lin, S. M. , X. M. Zhou , Y. L. Zhou , et al. 2020. “Intestinal Morphology, Immunity and Microbiota Response to Dietary Fibers in Largemouth Bass, *Micropterus salmoide* .” Fish and Shellfish Immunology 103: 135–142. 10.1016/j.fsi.2020.04.070. 32423866

[emi470438-bib-0049] Lin, X. , X. Zhou , Y. Lu , et al. 2025. “Safety Assessment and Probiotic Potential of a Novel Species *Lactobacillus xujianguonis* .” Nutrients 17, no. 21: 3474. 10.3390/nu17213474. PMC12610698 41228546

[emi470438-bib-0050] Liu, Q. , L. Wen , X. Pan , et al. 2021. “Dietary Supplementation of *Bacillus subtilis* and *Enterococcus faecalis* Can Effectively Improve the Growth Performance, Immunity, and Resistance of Tilapia Against *Streptococcus agalactiae* .” Aquaculture Nutrition 27, no. 4: 1160–1172. 10.1111/anu.13256.

[emi470438-bib-0051] Lorgen‐Ritchie, M. , T. Uren Webster , J. McMurtrie , et al. 2023. “Microbiomes in the Context of Developing Sustainable Intensified Aquaculture.” Frontiers in Microbiology 14: 1200997. 10.3389/fmicb.2023.1200997. PMC10327644 37426003

[emi470438-bib-0052] Maas, R. M. , Y. Deng , Y. Dersjant‐Li , et al. 2021. “Exogenous Enzymes and Probiotics Alter Digestion Kinetics, Volatile Fatty Acid Content and Microbial Interactions in the Gut of Nile Tilapia.” Scientific Reports 11, no. 1: 8221. 10.1038/s41598-021-87408-3. PMC8050056 33859242

[emi470438-bib-0053] Maas, R. M. , M. C. J. Verdegem , S. Debnath , L. Marchal , and J. W. Schrama . 2021. “Effect of Enzymes (Phytase and Xylanase), Probiotics ( *B. amyloliquefaciens* ) and Their Combination on Growth Performance and Nutrient Utilisation in Nile Tilapia.” Aquaculture 533: 736226. 10.1016/j.aquaculture.2020.736226.

[emi470438-bib-0054] MacWilliams, M. P. 2016. Indole Test Protocol. American Society for Microbiology. https://www.asmscience.org.

[emi470438-bib-0055] Mazanko, M. S. , E. V. Mazanko , S. A. Emelyantsev , S. V. Kozmenko , and D. V. Rudoy . 2025. “Evaluation of the Probiotic Potential of *Bacillus* spp. Strains Isolated From River Bottom Sediments.” Environments 13, no. 1: 13. 10.3390/environments13010013.

[emi470438-bib-0056] Melander, R. J. , M. J. Minvielle , and C. Melander . 2014. “Controlling Bacterial Behavior With Indole‐Containing Natural Products and Derivatives.” Tetrahedron 70, no. 37: 6363–6372. 10.1016/j.tet.2014.05.089. PMC4175420 25267859

[emi470438-bib-0057] Midhun, S. J. , S. Neethu , D. Arun , et al. 2019. “Dietary Supplementation of *Bacillus licheniformis* HGA8B Improves Growth Parameters, Enzymatic Profile and Gene Expression of *Oreochromis niloticus* .” Aquaculture 505: 289–296. 10.1016/j.aquaculture.2019.02.064.

[emi470438-bib-0058] Mirzabekyan, S. , N. Harutyunyan , A. Manvelyan , et al. 2023. “Fish Probiotics: Cell Surface Properties of Fish Intestinal Lactobacilli and *Escherichia coli* .” Microorganisms 11, no. 3: 595. 10.3390/microorganisms11030595. PMC10052099 36985169

[emi470438-bib-0059] Mitjana, O. , C. Bonastre , D. Insua , et al. 2014. “The Efficacy and Effect of Repeated Exposure to 2‐Phenoxyethanol, Clove Oil and Tricaine Methanesulphonate as Anesthetic Agents on Juvenile Angelfish ( *Pterophyllum scalare* ).” Aquaculture 433: 491–495. 10.1016/j.aquaculture.2014.07.013.

[emi470438-bib-0060] Mohamad, N. , H. Manan , M. Sallehhuddin , N. Musa , and M. Ikhwanuddin . 2020. “Screening of Lactic Acid Bacteria Isolated From Giant Freshwater Prawn ( *Macrobrachium rosenbergii* ) as Potential Probiotics.” Aquaculture Reports 18: 100523. 10.1016/j.aqrep.2020.100523.

[emi470438-bib-0061] Morelli, L. , and L. Capurso . 2012. “FAO/WHO Guidelines on Probiotics.” Journal of Clinical Gastroenterology 46: S1–S2. 10.1097/MCG.0b013e318269fdd5. 22955349

[emi470438-bib-0062] Mugwanya, M. , M. A. O. Dawood , F. Kimera , and H. Sewilam . 2022. “Anthropogenic Temperature Fluctuations and Their Effect on Aquaculture: A Comprehensive Review.” Aquaculture and Fisheries 7, no. 3: 223–243. 10.1016/j.aaf.2021.12.005.

[emi470438-bib-0063] Mujeeb Rahiman, K. M. , Y. Jesmi , A. P. Thomas , and A. A. Mohamed Hatha . 2010. “Probiotic Effect of *Bacillus* NL110 and *Vibrio* NE17 on the Survival, Growth Performance and Immune Response of *Macrobrachium rosenbergii* (de Man).” Aquaculture Research 41, no. 9: e120–e134. 10.1111/j.1365-2109.2009.02473.x.

[emi470438-bib-0064] Mukherjee, A. , and K. Ghosh . 2016. “Antagonism Against Fish Pathogens by Cellular Components and Verification of Probiotic Properties in Autochthonous Bacteria Isolated From the Gut of an Indian Major Carp, *Catla catla* (Hamilton).” Aquaculture Research 47, no. 7: 2243–2255. 10.1111/are.12676.

[emi470438-bib-0065] Muñoz‐Atienza, E. , B. Gómez‐Sala , C. Araújo , et al. 2013. “Antimicrobial Activity, Antibiotic Susceptibility and Virulence Factors of Lactic Acid Bacteria of Aquatic Origin Intended for Use as Probiotics in Aquaculture.” BMC Microbiology 13, no. 1: 15. 10.1186/1471-2180-13-15. PMC3574848 23347637

[emi470438-bib-0066] Nakharuthai, C. , S. Boonanuntanasarn , J. Kaewda , and P. Manassila . 2023. “Isolation of Potential Probiotic *Bacillus* spp. From the Intestine of Nile Tilapia to Construct Recombinant Probiotic Expressing CC Chemokine and Its Effectiveness on Innate Immune Responses in Nile Tilapia.” Animals 13, no. 6: 986. 10.3390/ani13060986. PMC10044694 36978530

[emi470438-bib-0067] Nayak, S. K. 2021. “Multifaceted Applications of Probiotic *Bacillus* Species in Aquaculture With Special Reference to *Bacillus subtilis* .” Reviews in Aquaculture 13, no. 2: 862–906. 10.1111/raq.12503.

[emi470438-bib-0068] Nayak, S. K. , and S. C. Mukherjee . 2011. “Screening of Gastrointestinal Bacteria of Indian Major Carps for a Candidate Probiotic Species for Aquaculture Practices.” Aquaculture Research 42, no. 7: 1034–1041. 10.1111/j.1365-2109.2010.02686.x.

[emi470438-bib-0109] Ng, W. , and C. Koh . 2017. “The Utilization and Mode of Action of Organic Acids in the Feeds of Cultured Aquatic Animals.” Reviews in Aquaculture 9, no. 4: 342–368. 10.1111/raq.12141.

[emi470438-bib-0069] Ntakirutimana, R. , K. M. Mujeeb Rahiman , A. A. Farhath , et al. 2026. “Host‐Derived Probiotics Outperform Commercial Formulations in Improving Growth, Gut Health and Disease Resistance in Red Tilapia.” Scientific Reports 16: 31678. 10.1038/s41598-026-58028-6. 42321300

[emi470438-bib-0070] Ntakirutimana, R. , K. M. Rahiman , J. Kunjappan , et al. 2025. “Dietary *Saccharomyces serevisiae* and *Pseudomonas aeruginosa* Enhance Growth Performance, Nutrient Utilization, Intestinal Morphology, and Hemato‐Biochemical Profile in Red Tilapia (*Oreochromis* sp.).” Probiotics and Antimicrobial Proteins 18: 6053–6068. 10.1007/s12602-025-10880-0. 41369877

[emi470438-bib-0071] Pereira, W. A. , C. M. N. Mendonça , A. V. Urquiza , et al. 2022. “Use of Probiotic Bacteria and Bacteriocins as an Alternative to Antibiotics in Aquaculture.” Microorganisms 10, no. 9: 1705. 10.3390/microorganisms10091705. PMC9503917 36144306

[emi470438-bib-0072] Pramanik, S. , S. Venkatraman , P. Karthik , and V. K. Vaidyanathan . 2023. “A Systematic Review on Selection Characterization and Implementation of Probiotics in Human Health.” Food Science and Biotechnology 32, no. 4: 423–440. 10.1007/s10068-022-01210-z. PMC9992678 36911328

[emi470438-bib-0073] Preetha, R. , S. Jose , S. Prathapan , et al. 2010. “An Inhibitory Compound Produced by *Pseudomonas* With Effectiveness on *Vibrio harveyi* .” Aquaculture Research 41, no. 10: 1452–1461. 10.1111/j.1365-2109.2009.02436.x.

[emi470438-bib-0074] Preetha, R. , K. K. Vijayan , N. S. Jayapraksh , S. V. Alavandi , T. C. Santiago , and I. S. Bright Singh . 2015. “Optimization of Culture Conditions for Mass Production of the Probiotics *Pseudomonas* MCCB 102 and 103 Antagonistic to Pathogenic Vibrios in Aquaculture.” Probiotics and Antimicrobial Proteins 7, no. 2: 137–145. 10.1007/s12602-015-9185-7. 25644493

[emi470438-bib-0075] Previti, A. , V. Biondi , F. Bruno , et al. 2025. “Text Mining and Topic Modeling Insights on Fish Welfare and Antimicrobial Use in Aquaculture.” BMC Veterinary Research 21, no. 1: 225. 10.1186/s12917-025-04544-y. PMC11956193 40165246

[emi470438-bib-0076] Rahayu, S. , K. Amoah , Y. Huang , et al. 2024. “Probiotics Application in Aquaculture: Its Potential Effects, Current Status in China and Future Prospects.” Frontiers in Marine Science 11: 1455905. 10.3389/fmars.2024.1455905.

[emi470438-bib-0077] Ringø, E. 2020. “Probiotics in Shellfish Aquaculture.” Aquaculture and Fisheries 5, no. 1: 1–27. 10.1016/j.aaf.2019.12.001.

[emi470438-bib-0078] Ringø, E. , H. Van Doan , S. H. Lee , et al. 2020. “Probiotics, Lactic Acid Bacteria and Bacilli: Interesting Supplementation for Aquaculture.” Journal of Applied Microbiology 129, no. 1: 116–136. 10.1111/jam.14628. 32141152

[emi470438-bib-0079] Rodrigues, P. M. , P. Ribeiro , and F. K. Tavaria . 2022. “Distinction of Different Colony Types by a Smart‐Data‐Driven Tool.” Bioengineering 10, no. 1: 26. 10.3390/bioengineering10010026. PMC9854692 36671597

[emi470438-bib-0080] Rwezawula, P. , W. Waiswa Mwanja , N. Vereecke , P. Bossier , and D. Vanrompay . 2025. “Advancing Aquaculture Probiotic Discovery via an Innovative Protocol for Isolation of Indigenous, Heat and Salt Tolerant, Quorum Quenching Probiotic Candidates.” Frontiers in Microbiology 16: 1558238. 10.3389/fmicb.2025.1558238. PMC12000936 40241725

[emi470438-bib-0081] Sabino, Y. N. V. , A. D. Paiva , B. R. Fonseca , J. D. Medeiros , and A. B. F. Machado . 2025. “Deciphering Probiotic Potential: A Comprehensive Guide to Probiogenomic Analyses.” Future Microbiology 20, no. 7–9: 611–622. 10.1080/17460913.2025.2492472. PMC12153390 40227157

[emi470438-bib-0082] Sachdeva, A. , T. Tomar , T. Malik , A. Bains , and A. Karnwal . 2025. “Exploring Probiotics as a Sustainable Alternative to Antimicrobial Growth Promoters: Mechanisms and Benefits in Animal Health.” Frontiers in Sustainable Food Systems 8: 1523678. 10.3389/fsufs.2024.1523678.

[emi470438-bib-0083] Sadeghi, A. , M. Ebrahimi , S. Shahryari , M. S. Kharazmi , and S. M. Jafari . 2022. “Food Applications of Probiotic Yeasts; Focusing on Their Techno‐Functional, Postbiotic and Protective Capabilities.” Trends in Food Science & Technology 128: 278–295. 10.1016/j.tifs.2022.08.018.

[emi470438-bib-0084] Sadeghi, M. , B. Panahi , A. Mazlumi , M. A. Hejazi , D. E. A. Komi , and Y. Nami . 2022. “Screening of Potential Probiotic Lactic Acid Bacteria With Antimicrobial Properties and Selection of Superior Bacteria for Application as Biocontrol Using Machine Learning Models.” LWT 162: 113471. 10.1016/j.lwt.2022.113471.

[emi470438-bib-0085] Saini, S. , R. Yadav , R. Das , and C. Singh . 2026. “Enzyme Therapy as a Promising Strategy to Overcome Multi‐Drug Resistance in Pathogens: Current Advances, Key Challenges, and Future Directions.” RSC Advances 16, no. 6: 5578–5605. 10.1039/D5RA06932G. PMC12833820 41602185

[emi470438-bib-0086] Sanders, M. E. , L. M. A. Akkermans , D. Haller , et al. 2010. “Safety Assessment of Probiotics for Human Use.” Gut Microbes 1, no. 3: 164–185. 10.4161/gmic.1.3.12127. PMC3023597 21327023

[emi470438-bib-0087] Sharma, A. , and H.‐J. Lee . 2025. “Antimicrobial Activity of Probiotic Bacteria Isolated From Plants: A Review.” Food 14, no. 3: 495. 10.3390/foods14030495. PMC11817414 39942088

[emi470438-bib-0088] Si, T. , A. Wang , H. Yan , et al. 2024. “Progress in the Study of Natural Antimicrobial Active Substances in *Pseudomonas aeruginosa* .” Molecules 29, no. 18: 4400. 10.3390/molecules29184400. PMC11434294 39339396

[emi470438-bib-0089] Siangpro, N. , S. Chuakrut , W. Sirimanapong , et al. 2023. “ *Lactiplantibacillus argentoratensis* and *Candida tropicalis* Isolated From the Gastrointestinal Tract of Fish Exhibited Inhibitory Effects Against Pathogenic Bacteria of Nile Tilapia.” Veterinary Sciences 10, no. 2: 129. 10.3390/vetsci10020129. PMC9958883 36851433

[emi470438-bib-0090] Simón, R. , F. Docando , N. Nuñez‐Ortiz , C. Tafalla , and P. Díaz‐Rosales . 2021. “Mechanisms Used by Probiotics to Confer Pathogen Resistance to Teleost Fish.” Frontiers in Immunology 12: 653025. 10.3389/fimmu.2021.653025. PMC8110931 33986745

[emi470438-bib-0091] Smith, C. A. , and A. M. Hussey . 2019. “Gram Stain Protocols.” https://www.asmscience.org.

[emi470438-bib-0092] Srirengaraj, V. , H. L. Razafindralambo , H. N. Rabetafika , H.‐T. Nguyen , and Y.‐Z. Sun . 2023. “Synbiotic Agents and Their Active Components for Sustainable Aquaculture: Concepts, Action Mechanisms, and Applications.” Biology 12, no. 12: 1498. 10.3390/biology12121498. PMC10740583 38132324

[emi470438-bib-0093] Standen, B. T. , D. L. Peggs , M. D. Rawling , et al. 2016. “Dietary Administration of a Commercial Mixed‐Species Probiotic Improves Growth Performance and Modulates the Intestinal Immunity of Tilapia, *Oreochromis niloticus* .” Fish and Shellfish Immunology 49: 427–435. 10.1016/j.fsi.2015.11.037. 26672904

[emi470438-bib-0094] Stasiak‐Różańska, L. , A. Berthold‐Pluta , A. S. Pluta , K. Dasiewicz , and M. Garbowska . 2021. “Effect of Simulated Gastrointestinal Tract Conditions on Survivability of Probiotic Bacteria Present in Commercial Preparations.” International Journal of Environmental Research and Public Health 18, no. 3: 1108. 10.3390/ijerph18031108. PMC7908519 33513771

[emi470438-bib-0095] Su, P.‐T. , C.‐T. Liao , J.‐R. Roan , S.‐H. Wang , A. Chiou , and W.‐J. Syu . 2012. “Bacterial Colony From Two‐Dimensional Division to Three‐Dimensional Development.” PLoS One 7, no. 11: e48098. 10.1371/journal.pone.0048098. PMC3498271 23155376

[emi470438-bib-0096] Tachibana, L. , G. S. Telli , D. d. C. Dias , et al. 2021. “ *Bacillus subtilis* and *Bacillus licheniformis* in Diets for Nile Tilapia ( *Oreochromis niloticus* ): Effects on Growth Performance, Gut Microbiota Modulation and Innate Immunology.” Aquaculture Research 52, no. 4: 1630–1642. 10.1111/are.15016.

[emi470438-bib-0097] Tennoune, N. , M. Andriamihaja , and F. Blachier . 2022. “Production of Indole and Indole‐Related Compounds by the Intestinal Microbiota and Consequences for the Host: The Good, the Bad, and the Ugly.” Microorganisms 10, no. 5: 930. 10.3390/microorganisms10050930. PMC9145683 35630374

[emi470438-bib-0098] Thi, Q. V. C. , T. Q. Dung , H. N. Hien , N. B. Trung , T. T. Dung , and N. P. Thuy . 2023. “Antibacterial Activity of Lactic Acid Bacteria From Various Freshwater Fish Species Against Pathogenic Bacteria in Caged Red Tilapia (*Oreochromis* sp.).” Biodiversitas 24, no. 6: 3373–3383. 10.13057/biodiv/d240633.

[emi470438-bib-0099] Tittsler, R. P. , and L. A. Sandholzer . 1936. “The Use of Semi‐Solid Agar for the Detection of Bacterial Motility.” Journal of Bacteriology 31, no. 6: 575–580. 10.1128/jb.31.6.575-580.1936. PMC543748 16559914

[emi470438-bib-0100] Treven, P. , D. Paveljšek , B. Bogovič Matijašić , and P. Mohar Lorbeg . 2024. “The Effect of Food Matrix Taken With Probiotics on the Survival of Commercial Probiotics in Simulation of Gastrointestinal Digestion.” Food 13, no. 19: 3135. 10.3390/foods13193135. PMC11475386 39410170

[emi470438-bib-0101] Vinoj, G. , B. Vaseeharan , B. DavidJayaseelan , P. Rajakumaran , and C. Ravi . 2013. “Inhibitory Effects of *Bacillus licheniformis* (DAB1) and *Pseudomonas aeruginosa* (DAP1) Against *Vibrio parahaemolyticus* Isolated From *Fenneropenaeus indicus* .” Aquaculture International 21, no. 5: 1121–1135. 10.1007/s10499-012-9617-2.

[emi470438-bib-0102] Wang, J. , D. Zhang , Y. Wang , Z. Liu , L. Liu , and C. Shi . 2021. “Probiotic Effects of the *Bacillus velezensis* GY65 Strain in the Mandarin Fish, *Siniperca chuatsi* .” Aquaculture Reports 21: 100902. 10.1016/j.aqrep.2021.100902.

[emi470438-bib-0103] Wang, L. , S. Ren , A. A. Behan , et al. 2025. “Probiotics and Their Antimicrobial Metabolites: A Collegial Strategy for Food Bio‐Preservation—A Review.” Food Science & Nutrition 13, no. 12: e71318. 10.1002/fsn3.71318. PMC12678059 41356233

[emi470438-bib-0104] Weisburg, W. G. , S. M. Barns , D. A. Pelletier , and D. J. Lane . 1991. “16S Ribosomal DNA Amplification for Phylogenetic Study.” Journal of Bacteriology 173, no. 2: 697–703. 10.1128/jb.173.2.697-703.1991. PMC207061 1987160

[emi470438-bib-0105] Yadav, R. , and P. Shukla . 2017. “An Overview of Advanced Technologies for Selection of Probiotics and Their Expediency: A Review.” Critical Reviews in Food Science and Nutrition 57, no. 15: 3233–3242. 10.1080/10408398.2015.1108957. 26505073

[emi470438-bib-0106] Yao, Y. , X. Wang , X. Lin , et al. 2024. “Isolation and Characterization of Probiotic Lysinibacillus Species From the Gastrointestinal Tract of Large Yellow Croaker ( *Larimichthys crocea* ).” Frontiers in Marine Science 11: 1408979. 10.3389/fmars.2024.1408979.

[emi470438-bib-0107] Yu, H. , S. Nazir , F. Ijaz , et al. 2025. “Dietary Supplementation of *Bacillus subtilis* as Probiotic Influenced the Growth Performance, Hematological Parameters, Immune Function, Antioxidant Status, and Digestive Enzyme Activity of Nile Tilapia Fingerlings ( *Oreochromis niloticus* ).” Animals 15, no. 9: 1256. 10.3390/ani15091256. PMC12071117 40362071

[emi470438-bib-0108] Yuan, Y. , Y. M. Yuan , Y. Dai , et al. 2022. “Preliminary Investigation on the Impact of Covid‐19 on Aquaculture in China: A Case Study on Farmed Tilapia and Channel Catfish Sector.” FAO Fisheries and Aquaculture Circular No. 1243. 10.4060/cb7868en.

