# Supplementary material for: Isolation, Characterization and Molecular Identification of Putative Probiotic Bacteria From Red Tilapia Gut
Source: Environ Microbiol Rep. 2026 Oct 21;18(5):e70438. doi: 10.1111/1758-2229.70438 (PMC13687586; doi:10.1111/1758-2229.70438)
Supplement: Supplementary file 1 [file EMI4-18-e70438-s001.docx]

## Isolation, characterization, and molecular identification of putative probiotic bacteria from red tilapia gut

Remy Ntakirutimana^a,b,d^*, Ashin Maria Jacob^b^, Aishath Ali Farhath^c,d^, Anatole Bigirimana^a,e^, N Regina Hershey ^c^, Megha Lovejan^b^, Paul Mwangi^f^, MP Jijina^b^, Fazeena Fariq^b^, C Varsha^b^, KM Mujeeb Rahiman^b^

^a^ Centre de Recherche en Sciences Naturelles et de l’Environnement (CRSNE), Universite du Burundi, PB 2700, Bujumbura, Burundi

^b^ School of Industrial Fisheries, Cochin University of Science and Technology, Kochi, Kerala, 682016, India.

^c^ Department of Marine Biology, Microbiology and Biochemistry, School of Marine Sciences, Cochin University of Science and Technology, Cochin 682016, Kerala, India

^d^ Maldives Wetlands Foundation, Malé Grand, 208, Malé City, Maldives

^e^ Department of Biology, Univerite du Burundi, PB 2700, Bujumbura, Burundi

^f^ Department of Biotechnology, Cochin University of Science and Technology, Kochi, Kerala, 682022, India.

Remy Ntakirutimana: Email: [ntare93@gmail.com](mailto:ntare93@gmail.com) ; ORCID: [0009-0002-5871-6063](javascript:popup_orcidDetail('https://orcid.org'%20,'0009-0002-5871-6063');)

Ashin Maria Jacob: Email: [ashinjessyjacob@gmail.com](mailto:ashinjessyjacob@gmail.com) ; ORCID: [0009-0008-4750-785X](https://orcid.org/0009-0008-4750-785X)

Aishath Ali Farhath: Email: [faraa.ali@gmail.com](mailto:faraa.ali@gmail.com) ; ORCID: [0009-0009-2004-8371](https://orcid.org/0009-0009-2004-8371)

Anatole Bigirimana: Email: [anatole.bigirimana@ub.edu.bi](mailto:anatole.bigirimana@ub.edu.bi) ; ORCID: [0000-0002-8167-3594](https://orcid.org/0000-0002-8167-3594)

N Regina Hershey: Email: [mail2reginahershey@gmail.com](mailto:mail2reginahershey@gmail.com) ; ORCID: [0000-0002-2700-8091](https://orcid.org/0000-0002-2700-8091)

Megha Lovejan: Email: [meghalovejan96@cusat.ac.in](mailto:meghalovejan96@cusat.ac.in) ; ORCID: [0000-0003-3387-5682](https://orcid.org/0000-0003-3387-5682)

Paul Mwangi: Email: [pauloficer83@gmail.com](mailto:pauloficer83@gmail.com) ; ORCID: [0009-0005-9825-2955](https://orcid.org/0009-0005-9825-2955)

MP Jijina: Email: [jijinaramachandran888@gmail.com](mailto:jijinaramachandran888@gmail.com) ; ORCID: [0009-0004-5116-1899](https://orcid.org/0009-0004-5116-1899)

Fazeena Fariq: Email: [phixa2020@gmail.com](mailto:phixa2020@gmail.com) ; ORCID: [0009-0004-4787-5266](https://orcid.org/0009-0004-4787-5266)

C Varsha: Email: [varshabalakrishnan920@gmail.com](mailto:varshabalakrishnan920@gmail.com) ; ORCID: -

KM Mujeeb Rahiman: Email: [rkmmujeeb@gmail.com](mailto:rkmmujeeb@gmail.com) ; ORCID: [0000-0003-3938-6206](https://orcid.org/0000-0003-3938-6206)

* **Corresponding author:**	Remy Ntakirutimana
 Email: [ntare93@gmail.com](mailto:ntare93@gmail.com)
 ORCID: [0009-0002-5871-6063](javascript:popup_orcidDetail('https://orcid.org'%20,'0009-0002-5871-6063');)

Centre de Recherche en Sciences Naturelles et de l’Environnement (CRSNE), Universite du Burundi, PB 2700, Bujumbura, Burundi

**Supplementary materials**

Table S 1 Morphometric characteristics of colonies isolated from red tilapia gut

| Isolates | Colony shape | Colour | Elevation | Surface | Diameter (mm) | Bacteria shape | Media |
| --- | --- | --- | --- | --- | --- | --- | --- |
| NA401 | Circular | Cream/Off-white | Convex | Smooth, moist | 2–3 | Rods | NA |
| NA402 | Irregular | Pale yellow | Raised | Rough/dull | 3–5 | Rods | NA |
| NA403 | Filamentous | White | Flat | Dry | 4–6 | Rods | NA |
| BA4P | Circular | Cream | Convex | Smooth, glistening | 2–4 | Long rods | BA |
| BA4B | Irregular | Dull white | Raised | Rough, dry | 3–5 | Rods | BA |
| BA4S | Spreading | Whitish | Flat | Matte, wrinkled | 5–8 | Rods | BA |
| PA4 | Irregular | Greenish-blue | Flat | Shiny, mucoid | 2–4 | Rods | CA |
| TSA40 | Circular | Yellowish-green | Low convex | Smooth | 2–3 | Rods | CA |
| TSA41 | Irregular | Light brown-green | Flat | Moist, translucent | 2–3 | Rods | CA |
| NA404 | Circular | Cream | Convex | Smooth edge | 2–3 | Rods | NA |
| NA4R | Irregular | Milky white | Raised | Rough surface | 3–5 | Rods | NA |
| NA4C | Circular | Pale yellow | Flat | Moist, smooth | 1–2 | Rods | NA |
| TSA401 | Circular | Beige | Convex | Smooth | 2–4 | Rods | TSA |
| TSA402 | Irregular | Cream | Flat | Dry | 3–5 | Rods | TSA |
| TSA403 | Irregular spreading | Yellow tinge | Raised | Shiny | 2–3 | Rods | TSA |
| NA4N | Circular | White | Convex | Smooth, glossy | 1–3 | Rods | NA |
| NA4S | Circular | Cream-yellow | Raised | Moist | 2–4 | Rods | NA |
| BA4G | Circular | Off-white | Raised | Dry, ground-glass | 3–7 | Large rods | BA |
| NA406 | Circular | Greenish | Low flat | Shiny, mucoid | 2–3 | Rods | CA |
| NA405 | Irregular | Light green | Flat | Translucent smooth | 2–4 | Rods | CA |
| TSA404 | Circular | Pale cream | Low convex | Smooth | 2–3 | Rods | TSA |
| TSA405 | Irregular | Off-white | Flat | Dry and dull | 3–4 | Rods | TSA |
| NA4M | Circular | White | Convex | Smooth | 2–3 | Rods | NA |
| NA4Y | Circular | Pale yellow | Raised | Moist | 2–4 | Rods | NA |
| BA4R | Rough spreading | Chalk-white | Raised | Dry, wrinkled | 5–8 | Rods | BA |
| NA4D | Circular | Cream | Low convex | Shining, sticky | 2–3 | Rods | NA |

Table S2 Screening of the antagonism activity of candidates with pathogens

|  | *A. hydrophila* | *S. agalactiae* | *S. iniae* |
| --- | --- | --- | --- |
| NA401 | - | - | - |
| NA402 | - | + | - |
| NA403 | - | - | - |
| BA4P | + | + | + |
| BA4B | - | - | - |
| BA4S | - | - | + |
| PA4 | + | + | + |
| TSA40 | - | - | - |
| TSA41 | - | - | + |
| NA404 | + | - | - |
| NA4R | - | - | - |
| NA4C | - | - | - |
| TSA401 | - | - | - |
| TSA402 | - | - | - |
| TSA403 | - | + | - |
| NA4N | - | - | - |
| NA4S | - | - | - |
| BA4G | + | + | + |
| NA406 | - | - | - |
| NA405 | - | - | - |
| TSA404 | - | - | - |
| TSA405 | - | - | - |
| NA4M | - | + | - |
| NA4Y | - | - | - |
| BA4R | - | - | - |
| NA4D | - | - | - |

Table S3 Tolerance to simulated gastrointestinal conditions (survival %)

| Parameter | PA4 | BA4P | BA4G | *p*-value |
| --- | --- | --- | --- | --- |
| pepsin | | | | |
| 0h | 100.00 ± 0.00ᵃ | 100.00 ± 0.00ᵃ | 100.00 ± 0.00ᵃ | NA |
| 1h | 83.00 ± 4.58ᵃ | 86.33 ± 1.53ᵃ | 84.67 ± 3.06ᵃ | 0.5058 |
| 4h | 78.67 ± 1.53ᵃᵇ | 81.67 ± 3.51ᵃ | 72.67 ± 3.21ᵇ | 0.0229 |
| 6h | 84.67 ± 2.52ᵃ | 84.33 ± 1.53ᵃ | 70.00 ± 3.61ᵇ | 0.0008 |
| pancreatin_bilesalts | | | | |
| 0h | 100.00 ± 0.00ᵃ | 100.00 ± 0.00ᵃ | 100.00 ± 0.00ᵃ | NA |
| 1h | 81.43 ± 2.92ᵃ | 83.36 ± 3.18ᵃ | 78.56 ± 1.10ᵃ | 0.15 |
| 4h | 78.50 ± 0.63ᵃ | 78.54 ± 1.28ᵃ | 73.31 ± 3.15ᵇ | 0.0288 |
| 6h | 83.09 ± 1.69ᵃ | 81.52 ± 2.13ᵃ | 72.63 ± 3.19ᵇ | 0.0038 |
| Hydrophobicity | 76.70 ± 3.50 | 78.16 ± 1.87 | 72.21 ± 1.69 | 0.0603 |

Values are mean ± SD. Different superscripts within the same row indicate significant differences among isolates (one-way ANOVA followed by Tukey’s HSD, p < 0.05).

Table S 4 Tolerance to variable salinity and temperature (Survival %)

| Parameter | PA4 | BA4P | BA4G | *p*-value |
| --- | --- | --- | --- | --- |
| temperature | | | | |
| 10°C | 46.78 ± 3.31ᵃ | 55.02 ± 2.55ᵃ | 49.74 ± 5.01ᵃ | 0.0901 |
| 20°C | 56.29 ± 5.90ᵇ | 86.05 ± 1.19ᵃ | 88.36 ± 5.05ᵃ | 0.0002 |
| 35°C | 97.59 ± 1.40ᵃ | 94.24 ± 3.05ᵃ | 96.56 ± 3.51ᵃ | 0.3857 |
| 40°C | 86.61 ± 2.10ᵃ | 88.71 ± 4.09ᵃ | 85.82 ± 1.93ᵃ | 0.4889 |
| salinity | | | | |
| 0%NaCl | 100.00 ± 0.00ᵃ | 100.00 ± 0.00ᵃ | 100.00 ± 0.00ᵃ | NA |
| 2% NaCl | 88.65 ± 1.22ᵃ | 89.09 ± 8.24ᵃ | 92.73 ± 4.54ᵃ | 0.6289 |
| 3% NaCl | 73.94 ± 1.43ᵇ | 79.07 ± 0.99ᵃ | 79.30 ± 1.25ᵃ | 0.0029 |

Values are mean ± SD. Different superscripts on the bars indicate significant differences among isolates (one-way ANOVA followed by Tukey’s HSD, p < 0.05).
